# From Molecules to Meaning: Integrating Neuropeptides, Sociostasis, and Hormesis in the Brain–Heart Axis

**DOI:** 10.3390/cimb48040386

**Published:** 2026-04-09

**Authors:** Hans P. Nazarloo, Stephen W. Porges, John M. Davis, C. Sue Carter

**Affiliations:** 1 Department of Psychiatry, College of Medicine-Jacksonville, University of Florida, 580 West 8th St., Tower II, 6th Floor, Jacksonville, FL 32209, USAcscarter@iu.edu (C.S.C.); 2 Department of Psychiatry, University of Illinois at Chicago, Chicago, IL 60607, USA; davisjm@uic.edu; 3 Traumatic Stress Research Consortium, Kinsey Institute, Indiana University, Bloomington, IN 47405, USA; 4 Department of Psychology, University of Virginia, Charlottesville, VA 22903, USA

**Keywords:** OT, VP, CRH, UCN, sociostasis, hormesis, brain, heart, stress

## Abstract

In an era marked by rising stress-related disorders and cardiovascular morbidity, understanding how the brain and heart adapt to environmental, physiological, and social stressors has become an urgent biomedical priority. This review advances an integrative framework centered on sociostasis, defined as the dynamic regulation of physiological state through social interaction, and its intersection with hormesis, a biphasic adaptive response to controlled stress that enhances resilience. We focus on four evolutionarily conserved neuropeptides, vasopressin, oxytocin, corticotropin-releasing hormone, and the urocortins, which serve as molecular bridges linking social behavior, neuroendocrine signaling, autonomic regulation, and cardiovascular function. Operating within an organized autonomic architecture, these systems calibrate responses to acute and chronic stress. Their context-dependent synergy enables adaptive flexibility under manageable challenge but may promote maladaptive cardiovascular remodeling when chronically dysregulated. Genetic vulnerability, developmental adversity, and persistent psychosocial stress can shift neuroendocrine–autonomic set points, increasing susceptibility to hypertension, endothelial dysfunction, and stress-induced cardiomyopathy. Conditioning and preconditioning paradigms illustrate how repeated exposure to subthreshold stressors primes cardiovascular tissues for future insults, enhancing ischemic tolerance and adaptive gene expression. We propose that cardiovascular hormesis depends not only on stimulus intensity but also on the integrity of neuroautonomic regulatory mechanisms that support recovery and flexibility. Vagal efficiency, a dynamic index of cardioinhibitory regulation, is discussed as a potential translational metric of adaptive capacity. By integrating molecular, physiological, and psychosocial perspectives, this framework conceptualizes cardiovascular resilience as an emergent property of coordinated hormetic signaling, neuropeptidergic modulation, autonomic regulation, and social buffering. Translational implications include peptide-based therapies, autonomic biofeedback, and behavioral interventions designed to enhance stress adaptability.

## 1. Hypotheses and an Overview of the Conceptual Framework

### 1.1. Stress Biology and the Social Determinants of Cardiovascular Disease

Despite advances in pharmacologic and procedural interventions, cardiovascular disease remains the leading cause of death and disability worldwide [1]. Its pathogenesis, traditionally attributed to lipid accumulation, mechanical strain, and genetic predisposition, is now recognized to be strongly influenced by stress biology and psychosocial factors [2,3]. Chronic caregiving stress elevates systolic blood pressure [4], while social isolation, comparable in mortality risk to smoking and obesity, independently predicts cardiovascular events [3,5].

These observations raise a central question: how do stress biology and social experience interact through neuroendocrine and autonomic mechanisms to shape cardiovascular outcomes? The autonomic nervous system (ANS) and hypothalamic–pituitary–adrenal (HPA) axis are primary mediators of the stress response [6,7]. Through rapid sympathetic activation and slower endocrine signaling, these systems alter cardiac output, vascular resistance, metabolism, and immune function. While acute responses can be adaptive [8,9], sustained or recurrent activation, particularly without buffering, promotes cardiovascular wear-and-tear, known as allostatic load [10,11,12,13].

### 1.2. Neuropeptides as Mediators of Brain–Heart Communication

Beyond classical stress pathways, a conserved group of neuropeptides, including oxytocin (OT), vasopressin (VP), corticotropin-releasing hormone (CRH), and urocortins (UCNs), plays a central role in coordinating brain–heart communication. These peptides act as molecular integrators linking limbic circuits of emotion and social behavior with autonomic and endocrine systems regulating cardiac and vascular function [14,15]. They respond not only to metabolic stress [16] but also to social cues such as touch, gaze, vocal prosody, and proximity, indicating that social experience is biologically embedded in cardiovascular regulation [17,18,19]. This forms the basis of sociostasis, defined as the dynamic regulation of physiological systems in response to social engagement and relational safety [20].

These neuropeptidergic systems operate within a hierarchically organized autonomic nervous system. Vagal cardioinhibitory pathways arise from two functionally distinct brainstem nuclei: the dorsal motor nucleus of the vagus (DMNX) and the nucleus ambiguus. The nucleus ambiguus generates myelinated ventral vagal fibers that coordinate cardiac regulation with social engagement behaviors, whereas DMNX pathways primarily regulate subdiaphragmatic organs and may mediate immobilization responses under extreme threat. This hierarchy provides a framework through which neuropeptides shape autonomic state, influencing whether responses favor recovery or defensive reactivity.

Unlike homeostasis, which maintains narrow set points, or allostasis, which reflects stress-induced recalibration [21], sociostasis is relational and adaptive, describing how social buffering modifies neural, hormonal, and autonomic responses [22,23,24]. Social connectedness is associated with lower sympathetic tone and higher heart rate variability (HRV). Increased high-frequency HRV (HF-HRV) reflects enhanced ventral vagal regulation indexed by respiratory sinus arrhythmia (RSA) and should not be conflated with the cholinergic anti-inflammatory pathway mediated by DMNX efferents [20,25,26]. Co-regulatory processes, such as synchronized physiology between partners or mother–infant dyads, are partly mediated by oxytocinergic and vagal pathways [27,28,29] and are associated with reduced blood pressure, improved vascular compliance, and enhanced myocardial recovery [30,31].

Conversely, social disruption, including loneliness, conflict, or bereavement, weakens these regulatory networks [32]. Loss of social buffering increases sympathetic tone, reduces OT signaling, and enhances CRH–UCN-driven arousal [33,34], contributing to impaired baroreflex sensitivity, endothelial dysfunction, atherosclerosis, and arrhythmia [35,36,37].

### 1.3. Sociostasis and Hormesis: A Dual Framework for Cardiovascular Resilience

Parallel to sociostasis, hormesis is a well-established principle in toxicology and systems biology that is increasingly recognized in cardiovascular physiology [38,39,40]. It describes a U-shaped dose–response in which low-level stressors activate adaptive pathways, whereas higher exposures are harmful [41]. In the cardiovascular system, this is exemplified by ischemic preconditioning, where brief sublethal ischemia induces protective signaling that reduces subsequent injury [42].

**Figure 1:**
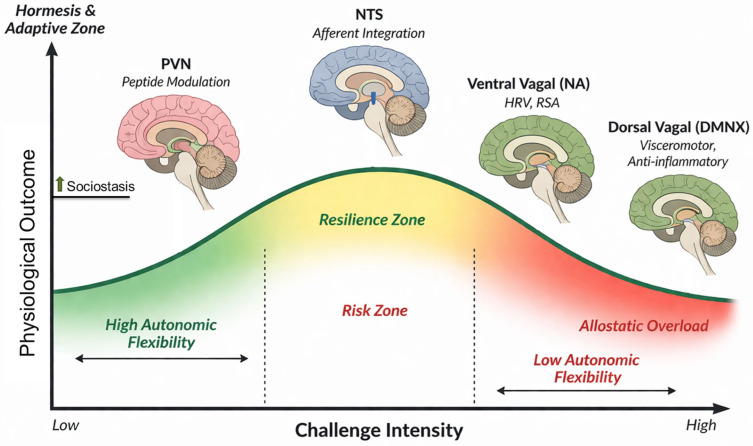
State-dependent hormesis and sociostasis in the brain–heart axis. It illustrates the relationship between challenge intensity (*x*-axis) and physiological outcome (*y*-axis), highlighting a central resilience zone where adaptive responses are optimized. At low-to-moderate challenge levels, high autonomic flexibility supports adaptive regulation and sociostasis. As challenge intensity increases beyond the optimal range, the system transitions into a risk zone, followed by allostatic overload, characterized by reduced flexibility and maladaptive physiological responses. Sociostasis acts as a contextual modifier that shifts baseline physiological state: increased sociostasis (↑) expands adaptive capacity, whereas reduced sociostasis constrains it. Key neuroanatomical structures are positioned along the curve to reflect their functional contributions across this continuum. The paraventricular nucleus (PVN) is associated with early peptide-mediated modulation under low challenge conditions. The nucleus tractus solitarius (NTS) represents central afferent integration at the peak of adaptive regulation. The ventral vagal complex (nucleus ambiguus; NA) supports parasympathetic regulation, including heart rate variability (HRV) and respiratory sinus arrhythmia (RSA), during the transition from adaptive to stress-responsive states. At high challenge intensity, the dorsal vagal complex (dorsal motor nucleus of the vagus; DMNX) predominates, contributing to visceromotor and anti-inflammatory responses associated with physiological shutdown and overload. This unified representation integrates physiological dynamics and neuroanatomical substrates within a shared coordinate framework, emphasizing how autonomic regulation shifts across increasing levels of stress and challenge.

Hormetic stimuli include moderate exercise, caloric restriction, thermal stress, and intermittent hypoxia [40,43]. Exercise increases OT and UCN levels and improves HRV. Increased HF-HRV reflects enhanced ventral vagal regulation indexed by RSA and should not be interpreted as direct activation of the dorsal vagal anti-inflammatory pathway [44]. These observations raise a key hypothesis: sociostasis and hormesis may function as interacting systems of cardiovascular resilience. Both engage neuropeptidergic and autonomic circuits to recalibrate cardiovascular responses [45], operate within controlled stress thresholds, and are context-dependent, varying with social buffering, prior stress exposure, and neuroendocrine state [46] (Figure 1).

Accordingly, the aim of this review is to integrate sociostasis and hormesis into a unified framework of cardiovascular resilience, focusing on how neuropeptidergic and autonomic mechanisms mediate the interaction between stress, social context, and cardiovascular function across varying environmental, genetic, and emotional conditions.

This interaction reveals a critical constraint: hormesis is not governed by stimulus intensity alone but by the state of the autonomic platform on which the stimulus is imposed. The capacity to benefit from mild challenges depends on the integrity of hierarchical autonomic regulation. When ventral vagal pathways originating in the nucleus ambiguus are functionally integrated with cardiopulmonary and cranial nerve circuits, physiological flexibility is preserved, allowing hormetic stimuli to promote repair, mitochondrial efficiency, and vascular resilience. However, when ventral vagal regulation is compromised and defensive states dominate, identical stimuli may be perceived as threatening, narrowing the adaptive window and accelerating inflammatory and metabolic dysregulation. Thus, hormesis in the brain–heart axis is inherently state-dependent.

When sociostasis and hormesis are absent or overwhelmed, due to early life adversity, chronic isolation, or persistent stress exposure, physiological rigidity, loss of autonomic flexibility, and disruption of peptidergic feedback loops occur [47]. These failures contribute to conditions such as Takotsubo cardiomyopathy [48], heart failure [49], stress-related arrhythmias, and hypertensive endothelial injury [50]. Such outcomes highlight the limitations of conventional risk models and underscore the need for a multidimensional framework integrating stress exposure, neuropeptide function, and social context [51]. Interventions aimed at restoring sociostasis, including structured social engagement, affective touch, emotional regulation training, and caregiving models, may re-establish ventral vagal–mediated regulation and reduce cardiovascular risk [52,53]. Similarly, hormetic strategies such as intermittent fasting, thermal therapy, and controlled hypoxia may enhance mitochondrial and vascular resilience [40,54]. Pharmacologic approaches targeting the OT–VP–CRH–UCN network, including intranasal OT, CRH agonists, UCN analogs, and VP receptor antagonists, offer additional avenues for modulating neurovisceral feedback in high-risk populations [55].

This review situates these concepts within a unified model in which cardiovascular health reflects the integration of physical, social, and molecular adaptability [56,57]. The neuropeptides OT, VP, CRH, and UCNs link environmental, emotional, and endothelial processes [15,58]. Their dysregulation signals impaired stress adaptation and disease progression [45,49], whereas their restoration may represent a promising therapeutic frontier in cardiovascular medicine [59].

### 1.4. Guiding Questions and Conceptual Scope

This review reframes cardiovascular adaptation and vulnerability through the integrated lens of sociostasis, hormesis, and neuropeptidergic modulation. It reflects the view that the heart is not merely a mechanical pump responding to hemodynamic cues, but a neurovisceral organ that integrates emotional, social, and metabolic signals through continuous interaction with the brain and environment [60]. Accordingly, the review addresses key questions that define this integrative framework (Table 1).

This table outlines the five central questions that structure the integrative framework linking sociostasis, hormesis, and neuropeptidergic modulation to cardiovascular adaptation and vulnerability. Each question addresses a distinct dimension, from molecular mechanisms to translational potential, highlighting the review’s focus on the heart as a neurovisceral organ shaped by continuous brain–environment interactions.

#### 1.4.1. Operational Definitions and State Dependence

In this review, challenges refer to any external perturbation (physical, metabolic, thermal, emotional, or social) capable of eliciting physiological change. Stress refers to the organism’s integrated neuroendocrine and autonomic response to that challenge, rather than the challenge itself. Hormesis is defined strictly as a biphasic, state-dependent biological response, wherein low-intensity challenges enhance adaptive capacity and resilience, while higher or poorly regulated exposures result in injury or dysfunction. Hormesis is not an intrinsic property of a stimulus, but an emergent property of the organism–environment interaction.

Allostasis describes adaptive recalibration of physiological systems in response to repeated challenges, whereas allostatic load reflects cumulative wear-and-tear when recovery is incomplete or regulatory flexibility is lost. Finally, sociostasis is defined here as the modulation of physiological state by social context, operating primarily through autonomic, neuropeptidergic, and inflammatory pathways. Sociostasis does not replace allostasis; rather, it shifts baseline state variables (e.g., vagal tone, HPA responsivity), thereby expanding or constraining the hormetic window. Throughout this review, high-frequency HRV (HF-HRV) is interpreted as an index of ventral vagal (nucleus ambiguus) cardioinhibitory regulation and not as a direct measure of dorsal vagal or cholinergic anti-inflammatory pathway activity.

#### 1.4.2. Lessons from the Conceptual History of Stress

Early physiological stress research highlighted a fundamental conceptual problem: stress was defined interchangeably as stimulus, organismal vulnerability, and physiological response, resulting in circular logic that obscured mechanism [61]. The same environmental condition could be debilitating in one individual yet adaptive or neutral in another, depending on physiological state at the time of exposure. This critique necessitated a reframing of stress as an interaction between challenge and organismal state. Hormesis risks repeating this historical error if low-dose challenges are labeled “hormetic” solely based on outcome, without reference to baseline physiological capacity. By explicitly distinguishing challenge, state, and response, and by incorporating autonomic efficiency metrics such as vagal efficiency (VE), the present framework avoids the circularity that previously limited progress in stress biology.

## 2. Hormesis: From Cellular Stress to Cardiovascular Systems Resilience

### 2.1. Molecular Mechanisms of Hormesis

Hormetic stimuli activate intracellular pathways including nuclear factor erythroid 2-related factor 2 (Nrf2), peroxisome proliferator-activated receptor gamma coactivator 1-alpha (PGC-1α), AMP-activated protein kinase (AMPK), sirtuins (SIRTs), and heat shock proteins, enhancing antioxidant defenses, mitochondrial function, angiogenesis, and anti-inflammatory signaling [62,63,64,65]. Hormesis describes a biphasic response in which low-intensity stressors induce adaptive mechanisms that promote cellular and systemic resilience. In the cardiovascular system, these responses support ischemic preconditioning, vascular repair, and improved stress tolerance during aging and metabolic dysfunction [66,67].

Oxidative Stress and ROS Signaling: Low levels of reactive oxygen species (ROS) act as signaling molecules in hormesis. Mitochondrial ROS activate redox-sensitive transcription factors such as Nrf2, which translocates to the nucleus and induces antioxidant response elements (AREs), upregulating genes including superoxide dismutase (SOD), glutathione peroxidase (GPx), catalase, and heme oxygenase-1 (HO-1) [68]. Nrf2 also attenuates inflammation by inhibiting NF-κB and reducing endothelial adhesion molecules and proinflammatory cytokines [69].

Heat Shock Proteins (HSPs): Thermal, oxidative, and metabolic stressors induce heat shock proteins (e.g., HSP70, HSP90), which function as molecular chaperones that preserve protein structure and prevent aggregation. HSP70 stabilizes mitochondrial membranes, modulates apoptotic signaling, and improves endothelial function during ischemia [70]. HSPs also interact with nitric oxide synthase (NOS) pathways, contributing to vasodilation and cardiomyocyte protection during reperfusion injury [71].

Autophagy and Mitophagy: Hormetic stimuli such as intermittent fasting, exercise, and caloric restriction activate autophagy, enabling degradation of damaged organelles and protein aggregates. Mitophagy selectively removes dysfunctional mitochondria, preserving bioenergetic efficiency and limiting ROS accumulation [72]. These processes are regulated by AMPK, sirtuins (notably SIRT1 and SIRT3), and PGC-1α, which coordinate mitochondrial biogenesis and antioxidant responses [73].

Collectively, these pathways illustrate that hormesis enhances resilience by promoting adaptive responses to transient stress rather than eliminating stress entirely. These conserved mechanisms operate across tissues, with significant implications for vascular function, cardiac excitability, and metabolic flexibility.

### 2.2. Hormetic Mediators Relevant to Cardiovascular Adaptation

At the cellular level, cardiovascular hormesis is mediated by a network of transcriptional regulators and signaling pathways that govern mitochondrial function, oxidative balance, and metabolic flexibility. Among the most important are PGC-1α, SIRT1, and Nrf2, which respond to sublethal stressors and support long-term cardiovascular protection.

PGC-1α is a key regulator of mitochondrial biogenesis and oxidative metabolism. It is upregulated by intermittent stressors such as exercise, hypoxia, and caloric restriction, leading to increased mitochondrial density, ATP production, and fatty acid oxidation in cardiac and vascular tissues [74]. These adaptations improve cardiac efficiency and resistance to ischemic and oxidative stress.

SIRT1, a NAD^+^-dependent deacetylase, functions as a metabolic sensor and stress-response regulator. It deacetylates PGC-1α, forkhead box O (FOXO) transcription factors, and endothelial nitric oxide synthase (eNOS), enhancing mitochondrial function, reducing endothelial inflammation, and increasing nitric oxide bioavailability. These effects support vascular tone and delay endothelial aging under stress conditions [68].

Nrf2 is a redox-sensitive transcription factor that regulates antioxidant and cytoprotective gene expression. In response to low-level oxidative stress, Nrf2 translocate to the nucleus and induces genes such as HO-1, GPx, and NAD(P)H quinone dehydrogenase 1 (NQO1), which reduce ROS, stabilize the endothelium, and limit vascular inflammation [75].

**Figure 2:**
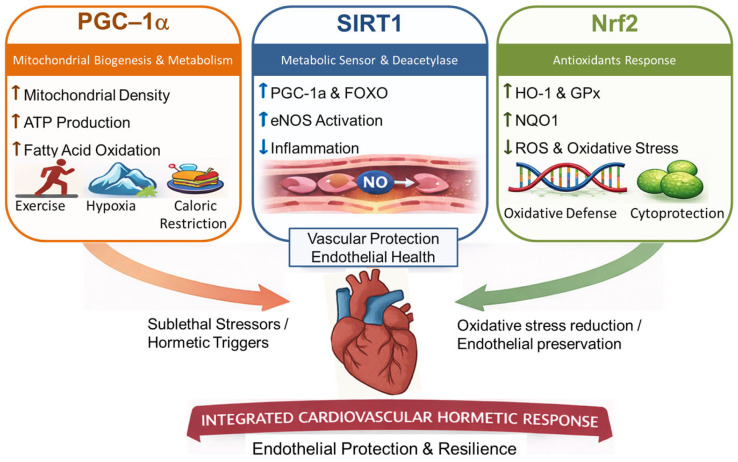
Hormetic mediators relevant to cardiovascular adaptation.

Collectively, these hormetic mediators form an adaptive signaling network that links subthreshold stress to cardioprotection. They exemplify how cellular and molecular resilience mechanisms are not only induced by physical stimuli like exercise or fasting but are also deeply intertwined with the molecular substrates of cardiovascular health (Figure 2).

Peroxisome proliferator-activated receptor gamma coactivator 1-alpha (PGC-1α), sirtuin 1 (SIRT1), and nuclear factor erythroid 2–related factor 2 (Nrf2) are key molecular mediators that link sublethal cellular stress to adaptive cardiovascular responses. PGC-1α regulates mitochondrial biogenesis and oxidative metabolism and is activated by exercise, hypoxia, and caloric restriction, thereby increasing mitochondrial density, Adenosine triphosphate (ATP) production, and fatty acid oxidation. The red arrow represents these sublethal stressors and hormetic triggers converging on the cardiovascular system. SIRT1 enhances mitochondrial and endothelial function through deacetylation of PGC-1α, forkhead box O (FOXO) transcription factors, and endothelial nitric oxide synthase (eNOS), thereby improving nitric oxide (NO) bioavailability and reducing inflammation. Nrf2 induces antioxidant and cytoprotective genes, including heme oxygenase-1 (HO-1), glutathione peroxidase (GPx), and NAD(P)H quinone oxidoreductase 1 (NQO1), leading to reduced reactive oxygen species (ROS) and oxidative stress. (↑) indicate increased expression, activation, or functional output of the indicated pathway or process, whereas (↓) indicate reduced inflammation, reactive oxygen species, or oxidative stress. The green arrow represents oxidative stress reduction and endothelial preservation in the cardiovascular system. Together, these pathways support endothelial protection and cardiovascular resilience.

### 2.3. Metabolic Sensors and Molecular Mediators of Hormetic Conditioning

Central to the hormetic adaptation are key molecular nodes:

AMP-activated protein kinase (AMPK): Senses intracellular ATP depletion and promotes metabolic reprogramming, autophagy, and endothelial resilience.

PI3K/Akt signaling: Mediates anti-apoptotic responses, enhances nitric oxide bioavailability, and supports mitochondrial recovery.

Heat shock proteins (HSP27, HSP70): Protect protein homeostasis, stabilize cytoskeletal structures, and confer resistance to ischemic and oxidative stress.

Taken together, these effectors function as integrative hubs, coordinating redox-sensitive, inflammatory, and metabolic responses central to cardio-protection.

### 2.4. Cardioprotective Hormetic Stimuli

Exercise and Shear Stress: Regular aerobic and resistance exercise induces hormetic adaptations by increasing vascular shear stress, enhancing nitric oxide bioavailability, and stimulating mitochondrial biogenesis. These effects are mediated in part by Nrf2 activation and upregulation of antioxidant enzymes, leading to improved endothelial function and myocardial resilience [75]. Exercise-induced increases in OT are associated with enhanced autonomic flexibility and baroreceptor sensitivity. Increases in HF-HRV reflect enhanced ventral vagal (nucleus ambiguus) cardioinhibitory control, rather than activation of the dorsal vagal cholinergic anti-inflammatory pathway, which represents a distinct visceromotor mechanism [76,77].

When autonomic flexibility is preserved, physiological or psychosocial challenges are more likely to elicit hormetic adaptation; under conditions of sympathetic dominance, inflammation, or impaired recovery, the same stimuli may become maladaptive.

Ischemic and Remote Ischemic Preconditioning: Ischemic preconditioning (IPC) and remote ischemic preconditioning (RIPC) involve brief, non-lethal ischemic episodes that protect against subsequent prolonged ischemia. These stimuli activate cardioprotective pathways, including mitochondrial ATP-sensitive potassium (K_ATP) channels, protein kinase C (PKC), and nitric oxide signaling, reducing infarct size and improving myocardial recovery. Clinical and translational studies support the efficacy of RIPC in attenuating perioperative myocardial injury and enhancing post-ischemic functional recovery [78,79].

Intermittent Fasting and Caloric Restriction: Intermittent fasting and caloric restriction elicit low-level metabolic stress that triggers autophagy, mitochondrial remodeling, and oxidative stress resistance. These adaptations involve AMPK-, SIRT1-, and PGC-1α-dependent signaling pathways and are associated with improved endothelial health, blood pressure regulation, and reduced atherosclerotic risk. Experimental models and human studies highlight their cardioprotective benefits, especially in aging and metabolic syndrome contexts [54].

**Figure 3:**
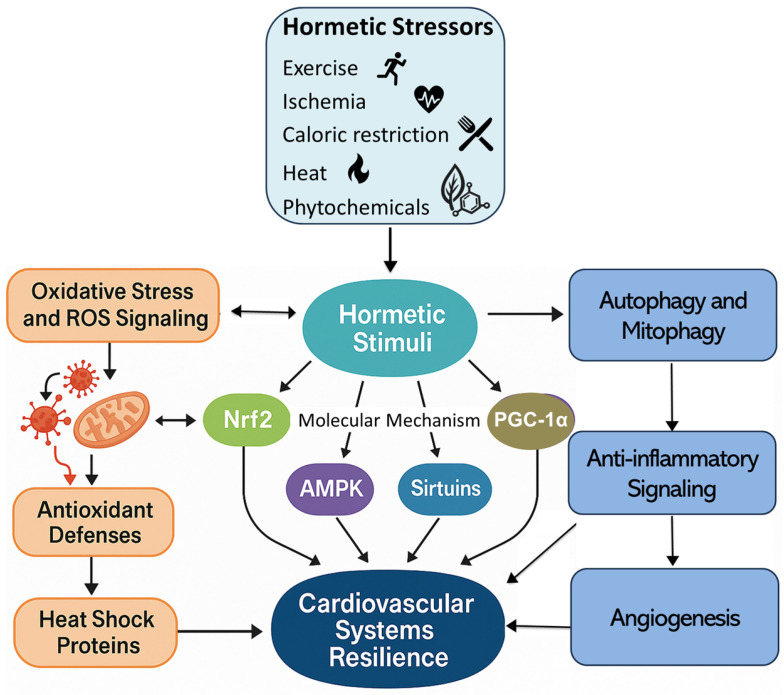
Hormesis: From Cellular Stress to Cardiovascular Systems Resilience. This schematic illustrates how low-intensity stressors initiate protective molecular responses that culminate in cardiovascular resilience. Hormetic stimuli, including exercise, ischemia, caloric restriction, heat, and phytochemicals, trigger mild oxidative stress and reactive oxygen species (ROS) signaling, which activate redox-sensitive pathways such as nuclear factor erythroid 2–related factor 2 (Nrf2), AMP-activated protein kinase (AMPK), sirtuins, and heat shock proteins (HSPs). Activation of Nrf2 promotes antioxidant defenses (e.g., superoxide dismutase [SOD], catalase, heme oxygenase-1), while AMPK and sirtuins coordinate mitochondrial biogenesis, autophagy, mitophagy, and metabolic adaptation. Concurrently, HSPs support protein folding and mitochondrial integrity under stress conditions. These integrated responses further engage anti-inflammatory signaling and promote angiogenesis, collectively enhancing endothelial function and reinforcing cardiovascular resilience against ischemic, oxidative, and metabolic challenges.

Thermal Stress: Sauna and Cold Plunge: Exposure to mild thermal stress, including sauna use and cold-water immersion, induces heat shock proteins (HSP70, HSP90), improves redox homeostasis, and enhances vascular compliance. Heat exposure lowers blood pressure, improves arterial stiffness, and promotes myocardial perfusion, whereas cold immersion improves sympathetic–parasympathetic balance. These hormetic effects are supported by recent cardiovascular physiology and thermoregulation studies [70] (Figure 3).

### 2.5. Conditioning and Preconditioning Concepts in Hormetic Cardiovascular Adaptation

Conditioning and preconditioning are key manifestations of hormesis in cardiovascular biology. These paradigms involve controlled exposure to sublethal stress that activates adaptive molecular and neuroendocrine processes, recalibrating response thresholds and enhancing resilience to subsequent insults. Ischemic preconditioning, consisting of brief intermittent ischemia, induces a biphasic protective response in the heart. Its efficacy depends not only on stimulus intensity but also on the physiological state in which it occurs, including the integrity of autonomic and cardiovascular regulatory mechanisms.

Early vs. late phase protection: The early phase develops within minutes and lasts 2–3 h, mediated by rapid post-translational modifications such as activation of protein kinase C (PKC), NOS, and mitochondrial ATP-sensitive potassium (K_ATP) channels. The late phase emerges after 12–24 h and persists up to 72 h, requiring transcriptional reprogramming and synthesis of cytoprotective proteins including heat shock protein 70 (HSP70), HO-1, and antioxidant enzymes [80,81]. Together, these phases illustrate temporally coordinated protective programs that enhance adaptive capacity.

Reprogramming of cellular stress response pathways: This biphasic response extends beyond the myocardium to the brain, vasculature, and kidney, reflecting systemic stress recalibration. Preconditioning promotes autophagy, improves mitochondrial dynamics, and restores redox balance through Nrf2 and AMPK-, SIRT1-, and PGC-1α-dependent signaling pathways [73]. Remote ischemic preconditioning, in which brief limb ischemia protects distant organs, further demonstrates that protective signals are transmitted via neural and humoral pathways, highlighting the coordinated nature of hormetic responses across organ systems [78].

Relevance to myocardial infarction, stroke, and aging: In aging, preconditioning can partially reverse mitochondrial dysfunction, improve endothelial function, and preserve myocardial contractility, thereby delaying heart failure and stroke vulnerability [54]. However, the magnitude of these benefits depends on baseline physiological state and regulatory capacity. These findings underscore the translational value of conditioning as both a mechanistic model and therapeutic strategy in age-related cardiovascular disease.

**Figure 4:**
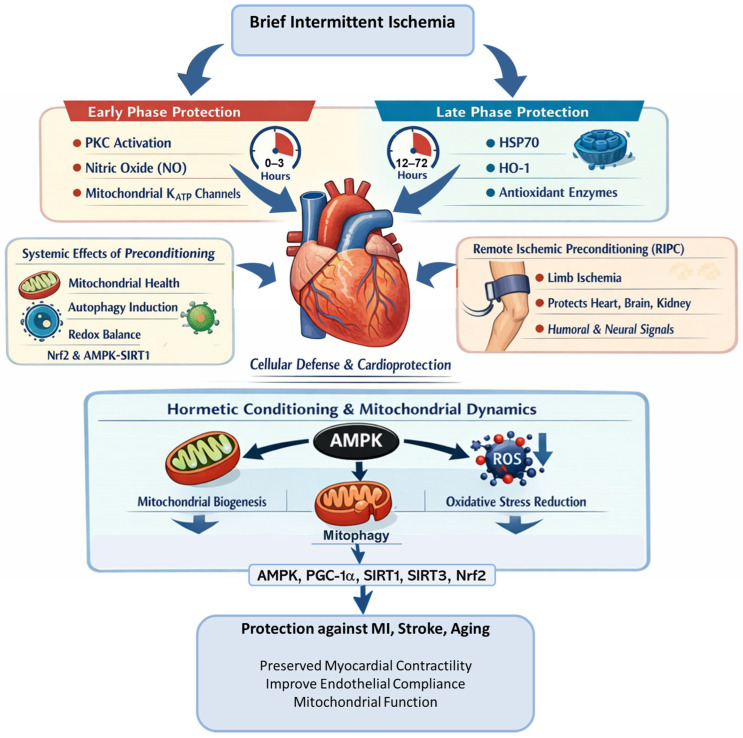
Conditioning and preconditioning as hormetic mechanisms of cardiovascular adaptation. Schematic summary of ischemic and hormetic conditioning pathways that confer cardioprotection through biphasic and systemic stress reprogramming. Brief ischemia–reperfusion cycles induce an early protective phase (minutes to ~2–3 h) mediated by rapid signaling events, including Protein kinase C (PKC) activation, nitric oxide (NO) production, and opening of mitochondrial K_ATP channels. A late (delayed) phase develops after ~12–24 h and persists up to 72 h, requiring transcriptional reprogramming and synthesis of cytoprotective proteins such as 70-kDa heat shock protein (HSP70), Heme oxygenase 1 (HO-1), and antioxidant enzymes. Conditioning effects extend beyond the heart to the vasculature, brain, and kidney via systemic recalibration of stress responses, involving improved mitochondrial function, autophagy, and redox balance through nuclear factor erythroid 2–related factor 2 (Nrf2)- and AMP-activated protein kinase (AMPK)–sirtuin 1 (SIRT1)–Peroxisome proliferator-activated receptor gamma coactivator 1-alpha (PGC-1α)–dependent pathways. Remote ischemic preconditioning (RIPC) transmits protection from limb ischemia to distant organs via humoral and neural signals. Repeated sublethal stimuli (e.g., exercise or intermittent ischemia) drive hormetic conditioning, enhancing mitochondrial biogenesis, mitophagy, and oxidative stress buffering, thereby promoting durable cardiovascular resilience.

Conditioning and preconditioning thus represent core mechanisms by which the cardiovascular system adapts to intermittent stress, reflecting hormesis as a context-sensitive adaptive response. These processes enhance myocardial and vascular resilience, but their effectiveness depends on the integrity of autonomic, neuroendocrine, and cellular regulation. In this context, conditioning expands the adaptive window in which physiological stress is translated into resilience rather than injury [82,83] (Figure 4).

#### 2.5.1. Ischemic Preconditioning and the Hormetic Reprogramming of Cardiovascular Tissues

Ischemic preconditioning (IPC) involves brief, non-lethal ischemia–reperfusion (I/R) cycles that activate a cascade of molecular signals, including reactive ROS as second messengers, PKC and phosphoinositide 3-kinase/protein kinase B (PI3K/Akt) pathways, mitochondrial ATP-sensitive potassium (mK_ATP) channels, and release of mediators such as adenosine, bradykinin, and opioids. These signals converge on pathways including mitogen-activated protein kinases (MAPKs), endothelial nitric oxide synthase (eNOS), and Nrf2, inducing cytoprotective gene expression, preserving mitochondrial integrity, reducing infarct size, and limiting post-ischemic apoptosis and inflammation [80,84,85,86].

#### 2.5.2. Exercise as a Physiological Hormetic Stimulus

Aerobic and resistance exercise are prototypical hormetic stimuli. Repeated increases in shear stress, ROS generation, and myocardial workload induce mitohormesis, vascular remodeling, and autonomic recalibration [63,87]. These adaptations include upregulation of PGC-1α, SIRT1, and SIRT3, increased eNOS activity and nitric oxide bioavailability, enhanced vasodilation, reduced arterial stiffness, and improved cardiac output [88,89,90]. Habitual exercise also reduces systemic inflammation and supports long-term cardiovascular resilience.

#### 2.5.3. Delayed and Remote Conditioning: Expanding Temporal and Spatial Dimensions

Delayed preconditioning, or second-window protection, occurs hours after the initial ischemic stimulus and involves transcriptional activation of protective genes, including heat shock proteins (e.g., HSP70), inducible nitric oxide synthase (iNOS), and antioxidant enzymes such as superoxide dismutase, catalase, and GPx [85,86].

Remote ischemic conditioning (RIC) extends this concept by inducing brief ischemia in a distant tissue, such as a limb, to protect organs including the heart and brain. RIC operates through humoral mediators (e.g., catecholamines, microRNAs), afferent neural pathways, and neuropeptides such as OT and VP, which contribute to systemic protective signaling [58,91,92]. These pathways activate remote organ responses and have demonstrated clinical benefit in myocardial infarction and cardiac surgery settings [93,94].

#### 2.5.4. Pharmacological Conditioning and Neuropeptide-Based Therapies

Pharmacological preconditioning aims to replicate endogenous protective signals using agents such as adenosine receptor agonists and mitochondrial K_ATP channel openers, as well as neuropeptides. OT administered prior to ischemia reduces infarct size, suppresses NF-κB–mediated inflammation, and promotes eNOS-dependent vasodilation via PI3K/Akt and ERK1/2 pathways [95,96,97]. Low-dose VP analogs enhance vascular tone and maintain hemodynamic stability through V1a receptor activation [98,99].

Postconditioning, initiated at reperfusion, reduces ROS burst, inhibits mitochondrial permeability transition pore (mPTP) opening, and limits neutrophil infiltration. OT and VP further mitigate reperfusion injury by stabilizing mitochondria, reducing calcium overload, and decreasing oxidative stress and inflammation [15,100].

#### 2.5.5. Hormetic Conditioning and Mitochondrial Dynamics

Repeated sublethal stress exposures, such as intermittent hypoxia, ischemia, or exercise, constitute hormetic conditioning, leading to cumulative cardiovascular adaptations [40,101]. These include mitochondrial biogenesis, endothelial progenitor cell mobilization, and reduced systemic inflammation [102].

At the cellular level, these adaptations involve enhanced mitochondrial biogenesis via PGC-1α and nuclear respiratory factors, increased mitophagy and quality control, and improved oxidative phosphorylation and ROS buffering [103]. These processes are regulated by sirtuins (SIRT1, SIRT3) and Nrf2, which coordinate nuclear–mitochondrial signaling essential for myocardial energetics and endothelial function [104].

Mitochondria serve as both sources and targets of ROS, central to cardiovascular hormesis. Subtoxic stress activates mitohormetic pathways that improve mitochondrial efficiency and quality control. OT and VP may further modulate these processes by influencing autonomic tone, redox signaling, and epigenetic regulation, reinforcing long-term adaptations [105].

#### 2.5.6. Integrating Neuropeptide Signaling into Conditioning Frameworks

The VP–OT axis provides a critical regulatory layer within conditioning paradigms. These neuropeptides coordinate autonomic balance, modulate inflammation and endothelial function, and influence mitochondrial redox signaling and gene expression. As such, they may enhance and prolong the effects of conditioning stimuli. Behavioral interventions, including social engagement and vagal stimulation, may augment OT release and amplify conditioning responses [105,106]. Across local, remote, pharmacologic, and systemic approaches, a shared principle emerges: sublethal stress induces a protective and anticipatory state.

**Figure 5:**
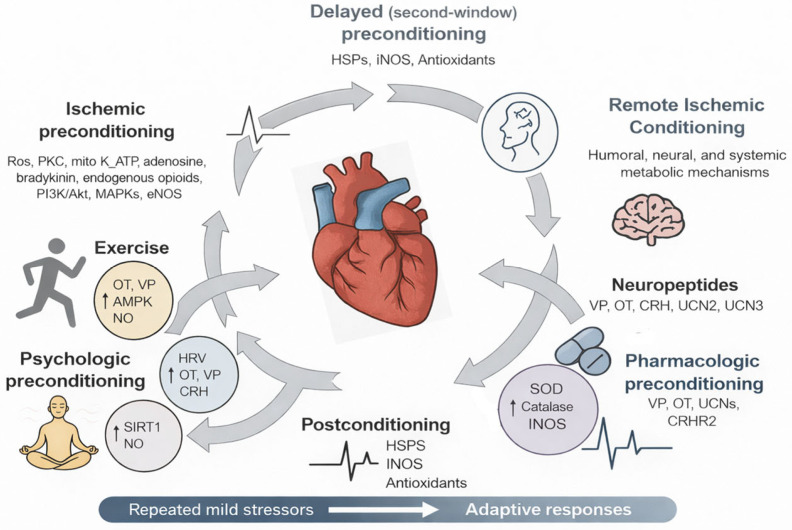
Hormetic Cardiovascular Adaptation via Conditioning and Preconditioning. This schematic illustrates how ischemic preconditioning (IPC), delayed (second-window) preconditioning, remote ischemic conditioning (RIC), exercise, psychological preconditioning, pharmacologic preconditioning, and postconditioning activate protective signaling pathways that enhance cardiovascular resilience. These pathways include reactive oxygen species (ROS) signaling, phosphoinositide 3-kinase/protein kinase B (PI3K/Akt), protein kinase C (PKC), AMP-activated protein kinase (AMPK), mitochondrial adaptation (including mitophagy, peroxisome proliferator-activated receptor gamma coactivator 1-alpha [PGC-1α], and sirtuin 1/3 [SIRT1/3]), and endothelial nitric oxide synthase (eNOS). Delayed preconditioning involves gene expression–dependent responses such as heat shock proteins (HSPs), inducible nitric oxide synthase (iNOS), and antioxidant systems. Psychological preconditioning engages neuroendocrine and autonomic pathways associated with heart rate variability (HRV) and modulation of oxytocin (OT), vasopressin (VP), and corticotropin-releasing hormone (CRH). Neuropeptides (OT, VP, CRH, urocortins [UCNs]) and antioxidant enzymes (e.g., superoxide dismutase [SOD], catalase) further support redox balance, anti-inflammatory signaling, and cellular protection. (↑) indicate increased expression, activation, or functional output of the indicated pathway. Large arrows indicate the directional flow and integration of conditioning pathways. Collectively, repeated mild stressors induce adaptive responses that enhance cardioprotection, myocardial energetics, and systemic resilience, providing a framework for therapeutic strategies targeting ischemia, inflammation, and heart failure.

Integrating neuropeptide signaling into conditioning strategies represents an emerging direction in cardioprotection. Targeted modulation of oxytocinergic and vasopressinergic systems may enhance the efficacy, durability, and systemic impact of conditioning protocols, offering new therapeutic avenues for cardiovascular diseases associated with ischemia, oxidative injury, and maladaptive remodeling (Figure 5).

### 2.6. Hormesis as a Dynamic Regulator of Autonomic and Vascular Tone

Baroreflex Sensitivity and Vagal Enhancement: Hormetic stimuli such as moderate exercise and controlled thermal exposure enhance baroreflex sensitivity and cardiac vagal regulation. Increased HF-HRV reflects enhanced ventral vagal (nucleus ambiguus) cardioinhibitory activity indexed by RSA and should not be conflated with DMNX cholinergic anti-inflammatory signaling. In a rodent model, eight weeks of moderate aerobic exercise restored age-related impairments in baroreflex gain and cardiac vagal modulation through neuroplastic changes in autonomic brain nuclei [44].

Hormetic Effects on Hierarchical Autonomic Regulation: Interventions such as exercise training and intermittent hypoxia improve hierarchical autonomic regulation, characterized by greater ventral vagal accessibility and reduced sympathetic dominance. In clinical populations, including individuals with diabetes, structured exercise improves HRV metrics (e.g., RSA, HF power) and reduces sympathetic predominance [107,108], consistent with enhanced ventral vagal regulation.

Microvascular Resilience and Endothelial Function: Low-dose hormetic interventions, including brief ischemia, heat exposure, and nutrient cycling, enhance endothelial resilience by increasing nitric oxide bioavailability, strengthening redox defenses, and preserving microvascular perfusion. Exercise and intermittent fasting improve flow-mediated dilation and microvascular integrity, particularly in aging populations [109]. These adaptations involve mitochondrial remodeling, heat shock protein induction, autophagy and mitophagy, and nitric oxide–mediated vascular protection. Conditioning paradigms provide both early (post-translational) and late (transcriptional) cytoprotection, improving ischemic tolerance, endothelial function, and myocardial recovery.

The capacity for hormetic adaptation depends on baseline autonomic and neuroendocrine state. Individuals with preserved ventral vagal regulation, high HRV, intact baroreflex sensitivity, and low inflammatory tone exhibit a broader hormetic window, enabling controlled stressors such as exercise, fasting, or thermal exposure to promote resilience. In contrast, sympathetic dominance, chronic inflammation, aging, trauma, or social isolation narrow this window, rendering identical stimuli maladaptive. Thus, hormesis reflects the interaction between stimulus intensity and organismal state. Foundational studies demonstrate that RSA reflects phasic vagal efferent modulation of sinoatrial node activity, providing the mechanistic basis for RSA–heart rate coupling [61,110].

#### Vagal Efficiency as a State-Dependent Index of Cardiovascular Hormesis

Vagal efficiency has emerged as a dynamic metric capturing the coupling between RSA and heart rate over short time windows. Defined as the slope of the RSA–heart rate relationship, VE reflects the functional accessibility and regulatory precision of ventral vagal (nucleus ambiguus) cardioinhibitory pathways in modulating sinoatrial node activity [111,112,113]. Unlike static HRV measures, which reflect variability amplitude, VE indexes the functional coupling between vagal output and cardiac slowing, reframing cardiovascular hormesis as a property of regulatory precision and organismal state rather than stimulus intensity alone [110,111,112,113].

Within a hierarchical autonomic framework, VE reflects the capacity of ventral vagal pathways to exert rapid inhibitory control over cardiac output in response to changing demands. High VE indicates efficient recruitment and withdrawal of vagal inhibition, supporting rapid recovery and cardiovascular flexibility, whereas reduced VE reflects diminished regulatory precision and adaptability. This metric is particularly relevant for hormesis because it quantifies the physiological substrate underlying adaptive stress responses. Individuals with higher VE exhibit more efficient cardioinhibitory control and faster recovery following perturbation, consistent with a broader hormetic window. Conversely, reduced VE identifies states in which similar challenges may exceed regulatory capacity and promote maladaptive inflammatory, metabolic, or vascular responses.

**Figure 6:**
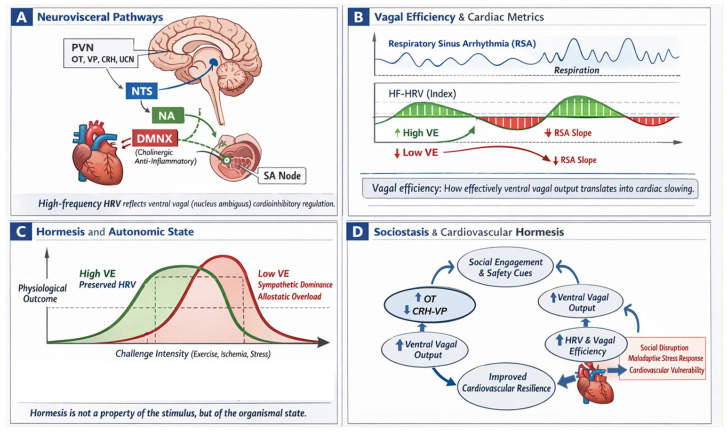
Vagal efficiency as a state-dependent regulator of cardiovascular hormesis within sociostasis. (**A**) Neurovisceral pathways linking brain and heart. Central neuropeptide systems originating in the paraventricular nucleus (PVN), including oxytocin (OT), vasopressin (VP), corticotropin-releasing hormone (CRH), and urocortin (UCN), converge on the nucleus tractus solitarius (NTS) and brainstem vagal nuclei. Ventral vagal efferents arising from the nucleus ambiguus (NA) provide myelinated cardioinhibitory control of the sinoatrial (SA) node, reflected in high-frequency heart rate variability (HRV), whereas dorsal motor nucleus of the vagus (DMNX) pathways primarily regulate visceromotor and cholinergic anti-inflammatory functions. The green dashed pathway denotes indirect or secondary vagal influences within the neurovisceral circuit, whereas the solid green pathway highlights the more direct ventral vagal (nucleus ambiguus) contribution to sinoatrial node control and cardiovagal regulation. (**B**) Vagal efficiency and cardiac metrics. Vagal efficiency (VE) indexes how effectively ventral vagal output translates respiratory-linked modulation into heart rate slowing. High VE is characterized by a steeper respiratory sinus arrhythmia (RSA) slope and preserved HF-HRV, whereas low VE reflects reduced coupling between respiration and cardiac chronotropy despite comparable respiratory input. (**C**) Hormesis and autonomic state. The physiological benefits of stressors or challenges (e.g., exercise, ischemia, psychosocial stress) depend on baseline autonomic organization. High VE preserves HRV across a broader hormetic window, supporting adaptive responses, whereas low VE is associated with sympathetic dominance, allostatic overload, and a narrowed or maladaptive hormetic range. This illustrates that hormesis is a property of organismal state rather than stimulus intensity alone. The green dotted lines delineate the optimal hormetic window, representing the range of challenge intensity within which high VE and preserved HRV support adaptive physiological outcomes. (↑) indicate increased expression, activation, or functional output of the indicated pathway or process, whereas (↓) indicate reduced expression and activation. (**D**) Sociostasis and cardiovascular hormesis. Social engagement and safety cues enhance ventral vagal output, increase HRV and vagal efficiency, and shift neuropeptide balance toward higher OT tone with reduced CRH–VP drive, collectively promoting cardiovascular resilience. In contrast, social disruption or chronic stress diminishes vagal efficiency, leading to maladaptive stress responses and increased cardiovascular vulnerability.

Conceptually, VE reframes cardiovascular hormesis as a function of neural regulatory precision and organismal state. Persistent reductions in VE may reflect long-term recalibration of autonomic set points following chronic stress or trauma, providing a measurable index of reduced adaptive capacity. Thus, VE serves as a translational bridge linking brainstem regulation, environmental context, and cardiovascular resilience (Figure 6).

## 3. Stress, Allostasis, and Disease Vulnerability

### 3.1. Biological and Psychosocial Stress

Stress, whether physiological or psychosocial, challenges hierarchical autonomic organization and regulatory stability. Acute stress responses are typically adaptive, mobilizing energy, enhancing alertness, and improving cardiovascular and cognitive performance through increased cardiac output and metabolic readiness. These responses are mediated by rapid autonomic nervous system (ANS) activation, particularly sympathetic signaling, and slower activation of the HPA axis, culminating in glucocorticoid release [114,115].

Biological stressors include trauma, infection, inflammation, ischemia, metabolic imbalance, and oxidative injury. Psychosocial stress arises from relational and societal contexts such as bereavement, caregiving, loneliness, early life adversity, social marginalization, financial insecurity, and perceived lack of control. Despite distinct origins, both engage overlapping neuroendocrine pathways and exert significant cardiovascular effects [116].

Psychosocial stress is often chronic and unpredictable. Limbic regions, including the amygdala, hippocampus, and prefrontal cortex, integrate these experiences and drive sustained neuroendocrine activation. Social isolation is associated with elevated cortisol, reduced HRV, increased sympathetic tone, and systemic inflammation, all contributing to cardiovascular risk [117]. Similarly, caregiving stress is linked to reduced vagal tone, elevated IL-6 and C-reactive protein (CRP), and endothelial dysfunction [72,118]. Socioeconomic deprivation and structural discrimination are associated with accelerated biological aging and arterial stiffness, consistent with the “weathering” hypothesis [119,120].

The cardiovascular system is particularly vulnerable to chronic stress due to its integration with autonomic and endocrine systems. Catecholamine surges promote vasoconstriction, platelet aggregation, and increased myocardial oxygen demand, while repeated sympathetic activation contributes to baroreflex dysfunction, arrhythmias, and hypertension. Chronic HPA activation impairs vascular repair and nitric oxide signaling, accelerating atherogenesis [48]. Animal models of social stress demonstrate structural cardiovascular changes, supporting a direct link between social context and disease [121].

Importantly, perceived threat alone can trigger these responses, highlighting the role of cognitive appraisal in shaping physiological outcomes. Interventions targeting emotional regulation, social connection, and cognitive reappraisal may therefore provide cardiovascular benefit. Protective factors such as perceived control, secure attachment, and social support buffer stress responses. Individuals with strong social support exhibit reduced cortisol reactivity, greater vagal flexibility, and improved vascular function under challenge [122,123,124].

From a hierarchical autonomic perspective, chronic stress reflects progressive impairment of ventral vagal regulation and narrowing of the adaptive window. Persistent sympathetic activation and reduced baroreflex sensitivity indicate disrupted brainstem-mediated cardioinhibitory control. Reduced VE may serve as an early marker of autonomic imprinting and diminished capacity to regulate cardiac output under stress. Chronic adversity may recalibrate autonomic set points, increasing susceptibility to arrhythmia, endothelial dysfunction, and stress-induced cardiomyopathy before overt disease becomes apparent.

### 3.2. Allostasis, Allostatic Load, and Wear-And-Tear

To maintain stability in changing environments, organisms rely on allostasis, defined as “stability through change” [125]. Unlike homeostasis, which maintains fixed set points, allostasis enables anticipatory adjustments in cardiovascular, neuroendocrine, immune, and metabolic systems in response to environmental demands [126]. These responses are mediated by the HPA axis, sympathetic nervous system, immune pathways, and metabolic hormones, which together increase alertness, mobilize energy, regulate vascular tone, and suppress non-essential functions [127]. While beneficial in acute settings, repeated or unresolved activation leads to dysregulation [128,129]. This maladaptive state, termed allostatic load, reflects the cumulative physiological burden of chronic stress [130,131]. It is characterized by persistent sympathetic activation, elevated cortisol, disrupted circadian rhythms, inflammation, and oxidative stress. These changes impair endothelial function, increase arterial stiffness, promote insulin resistance, and accelerate cellular aging [45,50].

Cardiovascular consequences include hypertension, cardiac hypertrophy, reduced HRV, and atherosclerosis. Chronic exposure to glucocorticoids and catecholamines promotes vascular remodeling, baroreceptor desensitization, and pro-arrhythmic myocardial activity [132]. Inflammatory mediators such as IL-6 and CRP further contribute to endothelial injury and thrombosis risk [133]. Individual variability in allostatic load reflects differences in stress exposure, psychosocial buffering, genetic factors, and developmental history. Early life adversity, for example, can recalibrate HPA and autonomic responses, a process described as biological embedding [134,135]. Allostatic load represents cumulative wear-and-tear on regulatory systems and provides a framework for understanding how chronic stress translates into cardiovascular risk and accelerated aging. Loss of flexibility in heart rate, vascular function, circadian rhythms, and neuroendocrine regulation is a hallmark of elevated allostatic burden and contributes to conditions such as heart failure with preserved ejection fraction (HFpEF), Takotsubo cardiomyopathy, and autonomic dysfunction [48].

Within a hierarchical autonomic framework, Takotsubo cardiomyopathy may reflect acute failure of ventral vagal regulation under extreme stress. Individuals with reduced VE may lack sufficient cardioinhibitory control to buffer sympathetic surges, rendering the myocardium vulnerable to catecholamine-mediated injury. In this context, Takotsubo represents not only sympathetic overactivation but a collapse of hierarchical autonomic regulation. Prospective VE assessment may help identify individuals at risk prior to clinical presentation.

From a hormetic perspective, elevated allostatic load reflects erosion of the adaptive window within which stress promotes resilience. As autonomic precision and recovery capacity decline, previously tolerable stressors become maladaptive. Thus, allostatic overload and loss of hormetic control represent converging manifestations of reduced regulatory flexibility across autonomic, vascular, and neuroendocrine systems.

### 3.3. Chronic Stress and Breakdown of Hormetic Control

Hormesis describes adaptive responses to low-dose, intermittent stress, but chronic or excessive stress disrupts this balance and erodes stress resilience. Under normal conditions, brief metabolic, thermal, or psychological challenges activate pathways such as Nrf2, SIRT1, AMPK, and PGC-1α, enhancing antioxidant defenses, mitochondrial efficiency, and anti-inflammatory signaling [62]. These responses support physiological flexibility and recovery from ischemic or inflammatory insults.

Chronic stress, however, impairs this adaptive machinery and promotes maladaptive remodeling. Sustained activation of the HPA axis and sympathetic nervous system leads to glucocorticoid resistance, catecholamine toxicity, and mitochondrial dysfunction [136]. At the vascular level, this results in reduced nitric oxide bioavailability, endothelial stiffening, and impaired flow-mediated dilation, markers of early vascular aging [71]. Key regulators of hormesis are suppressed under chronic stress. Nrf2 becomes uncoupled from redox signaling, limiting antioxidant responses [137]. PGC-1α, essential for mitochondrial biogenesis, is downregulated by persistent cortisol exposure and oxidative stress [78]. Similarly, SIRT1 activity declines in stressed vasculature, further reducing metabolic and endothelial resilience [73].

**Figure 7:**
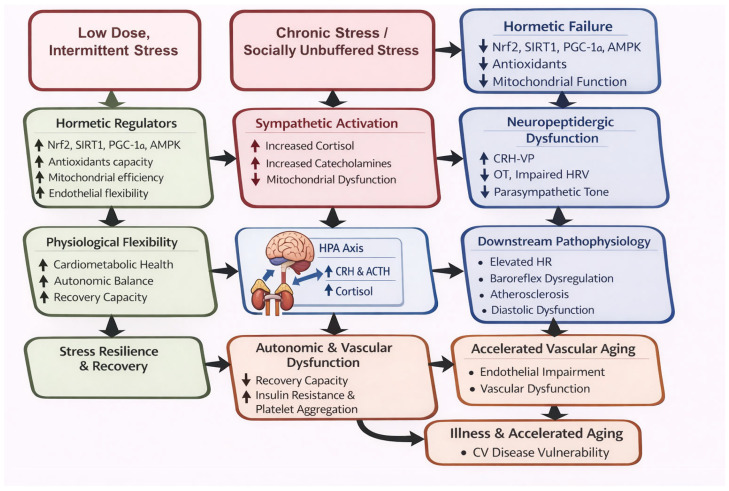
Divergent effects of adaptive versus chronic stress on hormetic control, neuroendocrine regulation, and cardiovascular aging. Low-dose, intermittent stress engages hormetic signaling pathways that enhance physiological flexibility and resilience. Under these conditions, key hormetic regulators, including peroxisome proliferator-activated receptor gamma coactivator 1-alpha (PGC-1α), sirtuin 1 (SIRT1), and nuclear factor erythroid 2–related factor 2 (Nrf2), and AMP-activated protein kinase (AMPK) are upregulated, promoting antioxidant capacity, mitochondrial efficiency, and endothelial flexibility. (↑) indicate increased expression, activation, or functional output of the indicated pathway or process, whereas (↓) indicate reduced expression and activation. This adaptive hormetic response supports dynamic physiological regulation, facilitating efficient stress recovery and long-term stress resilience.

Loss of hormetic control also disrupts autonomic regulation. Reduced baroreflex sensitivity, decreased HRV, and elevated sympathetic tone are linked to impaired stress adaptation and neuropeptidergic imbalance [44]. This combination of autonomic rigidity and vascular dysfunction creates a substrate for arrhythmogenesis, hypertension, and cardiac decompensation (Figure 7).

In contrast, chronic or socially unbuffered stress disrupts hormetic control and drives sustained sympathetic and HPA axis activation. Persistent elevations in catecholamines and cortisol contribute to mitochondrial dysfunction and metabolic strain, while prolonged corticotropin-releasing hormone (CRH)– adrenocorticotropic hormone (ACTH)–cortisol signaling exacerbates autonomic imbalance. Concurrent hormetic failure is characterized by downregulation of Nrf2-, SIRT1-, and PGC-1α–dependent protective mechanisms, leading to reduced antioxidant defenses and impaired mitochondrial function. Chronic stress further induces neuropeptidergic dysfunction, marked by increased CRH–Vasopressin (VP) signaling, reduced OT tone, impaired heart rate variability (HRV), and diminished parasympathetic activity. These changes culminate in autonomic and vascular dysfunction, including reduced recovery capacity, increased insulin resistance and platelet aggregation, baroreflex dysregulation, and elevated resting heart rate. Over time, these maladaptive processes promote downstream cardiovascular pathophysiology and accelerate vascular aging, manifesting as endothelial impairment, vascular dysfunction, and heightened risk for atherosclerosis and diastolic dysfunction.

Chronic stress may also impair OT, VP, and CRH/UCN signaling, key mediators of neurovisceral integration. Reduced oxytocinergic tone has been observed in social isolation and stress-related disorders such as post-traumatic stress disorder (PTSD) and depression [138,139]. Concurrently, sustained activation of CRH and UCN pathways without adequate social buffering may shift cardiovascular set points toward hypervigilance and inflammation [140]. Inflammation and autonomic dysregulation form a reinforcing loop, where inflammatory signaling increases sympathetic output, further sustaining inflammatory tone.

Thus, erosion of hormetic regulation under chronic stress reflects a systemic breakdown of interconnected neuroendocrine, vascular, and mitochondrial networks. The resulting cardiovascular state is characterized by reduced flexibility, persistent inflammation, and impaired energy metabolism, increasing vulnerability to adverse events and limiting response to conventional therapies. Repeated or unbuffered stress exposures may induce durable recalibration of hierarchical autonomic regulation. Consistent with the functional differentiation of ventral and dorsal vagal pathways, this shift can be conceptualized as an autonomic imprint, marked by reduced ventral vagal flexibility and increased susceptibility to sympathetic or dorsal vagal dominance. In some cases, acute overwhelming events may act as sentinel trauma, abruptly altering autonomic set points and VE over time. Such imprinting reshapes how hormetic stimuli are processed, linking early adversity and chronic stress to long-term cardiovascular risk. Restoring hierarchical autonomic regulation through social reconnection, behavioral conditioning, and targeted pharmacologic strategies therefore represents a critical therapeutic goal.

### 3.4. Peptidergic Dysregulation in Prolonged Stress

Neuropeptidergic systems, including OT, VP, CRH, and urocortins (UCNs), are central to stress adaptation and recovery. Under acute conditions, these peptides regulate cardiovascular tone, autonomic balance, immune activity, and metabolism. Chronic or unbuffered stress, however, disrupts this regulatory network, leading to systemic dysfunction.

Oxytocin System Downregulation: Prolonged stress, particularly with social isolation or trauma, reduces OT release, promotes receptor desensitization, and impairs social buffering [141,142]. This diminishes parasympathetic regulation and coordinated cardiovascular rhythms, increasing susceptibility to hypertension and arrhythmia.

Vasopressin and CRH Hyperactivation: Chronic stress enhance VP and CRH signaling, driving sympathetic activation, adrenocorticotropic hormone (ACTH) release, and glucocorticoid output. Sustained CRH activity promotes neuroinflammation, endothelial dysfunction, and autonomic rigidity [143,144,145]. Elevated VP, particularly via V1a receptors, contributes to vasoconstriction, platelet aggregation, and cardiac remodeling [58,146]. This shift toward CRH–VP dominance represents a key neuroendocrine transition in stress-related cardiovascular disease.

Urocortins and CRHR2 Desensitization: UCNs typically exert cardioprotective and anti-inflammatory effects via CRHR2. Under chronic stress and inflammation, CRHR2 signaling may become desensitized, reducing these protective effects [147]. This loss has been associated with impaired myocardial recovery, endothelial dysfunction, and reduced stress tolerance.

Functional Consequences of Peptidergic Imbalance: The combined effects of reduced OT signaling, increased VP and CRH activity, and impaired UCN–CRHR2 function lead to disrupted neurovisceral coordination. Autonomic regulation becomes rigid, cardiovascular responses become exaggerated, and cumulative tissue damage occurs in the heart, vasculature, and kidneys [148]. These mechanisms contribute to conditions such as Takotsubo cardiomyopathy, HFpEF, atherosclerosis, and neurocardiogenic syncope [48,49]. Restoring neuropeptidergic balance through behavioral interventions, targeted pharmacology, and social engagement represents a promising therapeutic strategy.

## 4. Sociostasis: Social Homeostasis in Health and Disease

### 4.1. Origin and Definition of Sociostasis

Regulation of internal stability is a core principle in physiology, traditionally framed through homeostasis and allostasis. Both models, however, focus on the individual organism as the unit of regulation. Sociostasis extends these frameworks by incorporating the regulatory influence of social relationships on physiology. Rooted in behavioral biology and developed within neuroscience, sociostasis proposes that social bonds act as external modulators of internal homeodynamic states [22,149,150].

This concept is not merely metaphorical. In socially evolved species, including rodents, non-human primates, and humans, physiological systems are closely linked to the presence or absence of familiar others. These interactions extend beyond behavior to influence neuroendocrine, cardiovascular, and autonomic outputs.

#### 4.1.1. Sociostasis: A Co-Regulatory Framework

Sociostasis describes a dynamic process through which social signals modulate physiological state, buffering threat and promoting safety. Signals such as touch, mutual gaze, vocal prosody, and movement synchrony are processed by neural systems that integrate sensory input with limbic, autonomic, and hormonal responses [151,152,153]. This framework recognizes that physiological stability is co-regulated across individuals, particularly in close relationships such as parent–child, romantic, and social bonds [117].

Empirical studies show that supportive social presence can reduce cortisol responses, enhance vagal tone, and increase activity in brain regions associated with safety and reward [154,155]. Conversely, social isolation or rejection disrupts these systems, increasing sympathetic tone, reducing OT signaling, and elevating inflammatory markers [48,149,156].

#### 4.1.2. Evolutionary and Developmental Foundations

Sociostasis has deep evolutionary origins. In altricial species, offspring depend on parental care not only for survival but also for physiological regulation. Maternal proximity regulates body temperature, heart rate, cortisol rhythms, and gene expression through epigenetic mechanisms [157,158]. These processes are mediated in part by OT and VP, which support bonding while modulating autonomic and cardiovascular function [19,150,159].

These mechanisms persist into adulthood through affiliative interactions, extending beyond kin relationships and reflecting a conserved neurobiological system that supports cooperative survival.

#### 4.1.3. From Social Behavior to Physiological Regulation

Sociostasis bridges psychological and biological domains by emphasizing that relational environments directly shape physiological function. Caregiving, attachment, and social exclusion influence heart rate, blood pressure, respiration, hormonal activity, and immune responses in real time [160]. These effects are not secondary to stress regulation but are integral to it, making sociostasis a key determinant of resilience [161,162].

This framework has important implications for modern populations. Social marginalization related to race, disability, or socioeconomic status contributes to physiological dysregulation not only through resource limitations but also through chronic social threat. Such exposures impair neuroendocrine regulation across the lifespan and increase risk for hypertension, metabolic syndrome, and inflammatory disease [163].

#### 4.1.4. Operationalizing Sociostasis

Sociostasis can be operationalized as the capacity of social context to modulate baseline autonomic and neuroendocrine states. Measurable indicators include increased high-frequency HF-HRV and VE, improved baroreflex sensitivity, reduced cortisol reactivity, decreased inflammatory markers (e.g., IL-6, C-reactive protein), and enhanced recovery following stress. Experimental approaches include controlled social presence, dyadic interaction paradigms, caregiving interventions, and social isolation models, enabling causal investigation of social influences on physiological resilience.

### 4.2. Dyadic and Group-Level Regulation of Physiology

Classical physiology has focused on individual homeostasis, yet growing evidence shows that physiological regulation is often shared across individuals. Dyadic regulation refers to co-regulation between two individuals in close relationships such as parent–child, romantic partners, or caregiver–patient dyads [164]. Group-level regulation extends this concept to larger social networks, where emotional contagion, behavioral synchrony, and shared environments influence health outcomes [165,166]. These dynamics highlight how social context shapes biological function.

#### 4.2.1. Dyadic Co-Regulation

Dyadic interactions characterized by trust, emotional closeness, or proximity can synchronize neural, hormonal, and autonomic activity. Hyperscanning EEG and fMRI studies demonstrate real-time neural synchrony between partners during shared tasks or emotional engagement [167,168]. Similarly, heart rate and respiratory rhythms can align during caregiving, rituals, or physical contact [29]. These synchronizations are associated with reduced cortisol, increased vagal tone, and improved immune function [169].

In maternal–infant dyads, skin-to-skin contact promotes synchrony in OT release, HRV, and thermoregulation, with lasting effects on stress regulation and attachment [170,171]. In romantic relationships, physical touch during stress reduces amygdala reactivity and sympathetic activation, supporting the concept that physiological safety is socially mediated [172].

#### 4.2.2. Neuroendocrine Coupling and Social Transmission

Dyadic regulation is mediated in part by shared neuropeptidergic signaling. OT facilitates social attunement and bonding while dampening threat circuitry and enhancing vagal regulation [150,152,173]. VP also contributes, particularly in male bonding and social vigilance, although its effects are more context dependent. These peptides interact with CRH–UCN and dopaminergic systems to regulate arousal, pain, and cardiovascular tone [174].

Neuroendocrine states can also be socially transmitted. Exposure to stressed individuals can induce sympathetic activation or cortisol elevation in observers [175]. Conversely, calm individuals can serve as “regulatory anchors,” reducing perceived threat and buffering physiological responses within social groups [142].

#### 4.2.3. Group-Level Synchrony and Physiological Outcomes

At the group level, physiological coupling occurs during collective rituals, synchronized movement, and shared emotional experiences. These states are associated with increased HF-HRV, elevated OT, and improved pain tolerance, reflecting group cohesion and shared meaning [176,177].

Social structure also influences health. In primates, social instability is linked to elevated cortisol, inflammation, and cardiovascular risk, whereas stable affiliative bonds promote resilience and longevity [178]. Similar patterns are observed in humans, where low social support or conflictual environments increase allostatic load and disease risk [51].

### 4.3. Role of OT and Social Interactions in Buffering Stress

Oxytocin, synthesized primarily in the paraventricular nucleus (PVN) and supraoptic nucleus (SON) of the hypothalamus, plays a central role in mediating the protective effects of social relationships on stress physiology. Beyond its roles in parturition and lactation, OT regulates emotional, autonomic, and cardiovascular function, particularly during social engagement and bonding [28,150,179].

#### 4.3.1. Central OT and Social Buffering of Stress

Central OT release is triggered by positive social interactions such as touch, eye contact, and verbal reassurance, serving as a key mediator of social buffering. OT modulates brain regions involved in threat processing, including the amygdala, bed nucleus of the stria terminalis (BNST), and ventromedial prefrontal cortex (vmPFC), reducing HPA axis activation and sympathetic output [180,181,182].

Experimental studies show that intranasal OT reduces cortisol responses to psychosocial stress, enhances emotion recognition, and increases trust-related behaviors [183,184]. In both animals and humans, OT supports pair bonding, maternal care, and social memory, processes closely linked to autonomic and cardiovascular regulation [150,185].

#### 4.3.2. OT, Vagal Function, and HRV

OT also modulates peripheral autonomic function. OT-producing neurons in the PVN project to the dorsal vagal complex, including the nucleus ambiguus and nucleus tractus solitarius (NTS), key centers for cardiac vagal control [173,186,187]. These pathways underpin the association between OT and increased HRV.

**Figure 8:**
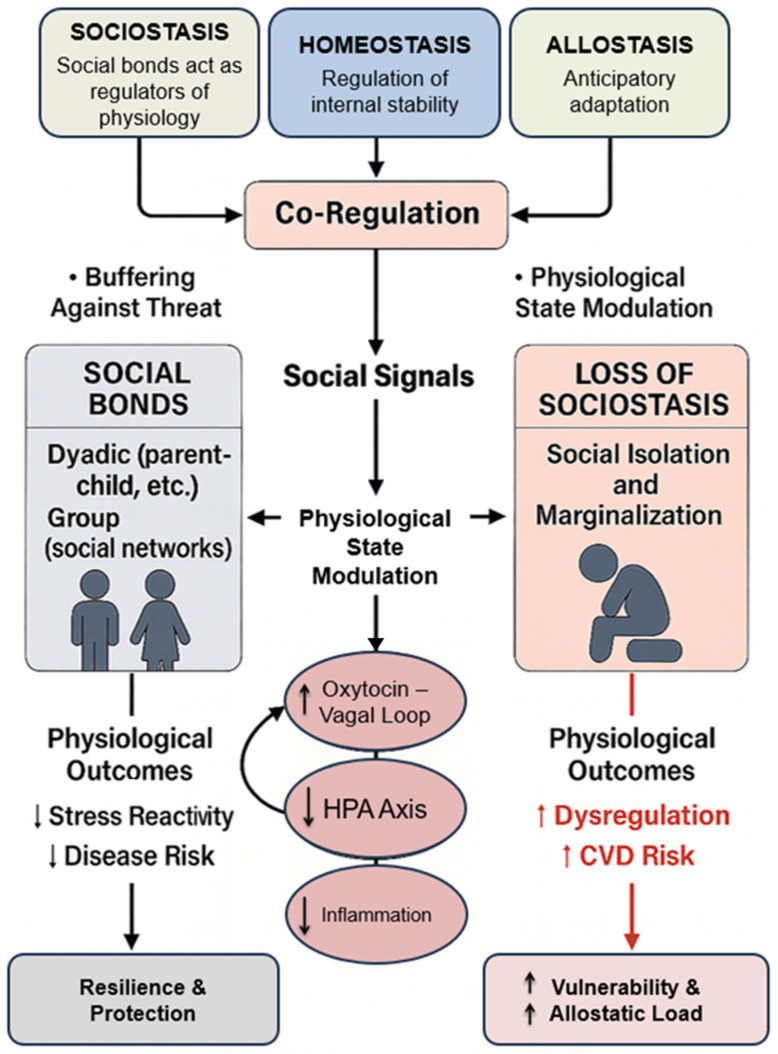
Sociostasis and physiological regulation. The diagram illustrates how homeostasis, allostasis, and sociostasis interact to shape stress responses and health outcomes. Social bonds support co-regulatory frameworks that enhance oxytocin (OT)–vagal function, reducing hypothalamic–pituitary–adrenal (HPA) activation, inflammation, and stress reactivity. In contrast, loss of sociostasis leads to elevated allostatic load, autonomic imbalance, and increased cardiovascular risk. (↑) indicate increased activation, or functional output of the indicated pathway or process, whereas (↓) indicate reduced activation, or functional output of the indicated pathway or process.

Increases in HF-HRV reflect enhanced ventral vagal (nucleus ambiguus) cardioinhibitory regulation indexed by RSA, rather than activation of dorsal vagal cholinergic anti-inflammatory pathways mediated by DMNX efferents [52,76]. Elevated OT levels are associated with higher HF-HRV, improved baroreflex sensitivity, and greater autonomic flexibility, helping explain the cardiovascular benefits of social connection and the risks associated with isolation (Figure 8).

#### 4.3.3. Clinical and Translational Relevance

Clinically, reduced OT levels are observed in individuals with early life adversity, chronic isolation, or PTSD, paralleling reductions in HRV and increased cardiovascular risk [188,189]. Conversely, caregiving, emotional closeness, and physical touch are associated with increased OT and improved stress biomarkers.

A randomized trial demonstrated that structured caregiving interventions increased urinary OT, improved HRV, and reduced endothelial dysfunction in at-risk caregivers [52]. These findings highlight the therapeutic potential of targeting OT pathways through behavioral interventions, social engagement, and pharmacologic approaches such as intranasal OT or OTR modulators to enhance stress resilience and cardiovascular health.

### 4.4. Vagal Pathways and HRV in Social Engagement

The vagus nerve serves as a primary anatomical and functional bridge between the brain and the cardiovascular system, mediating parasympathetic regulation of heart rate, inflammatory tone, and social behavior. Social engagement activates ventral vagal (nucleus ambiguus) cardioinhibitory pathways, supporting physiological calm, emotional regulation, and cardiovascular resilience. This ventral vagal activity is indexed by HF-HRV and RSA and should be distinguished from DMNX pathways involved in visceromotor and cholinergic anti-inflammatory control [180,189,190,191].

#### 4.4.1. The Polyvagal Theory and Social Engagement System

Polyvagal Theory describes a hierarchical autonomic organization in which the myelinated ventral vagal complex supports the social engagement system. This system enables suppression of defensive responses and promotes social interaction through coordinated facial expression, vocal prosody, and cardiac regulation [27,153,186,189,192,193]. Ventral vagal activation facilitates cardioinhibitory control, respiratory–cardiac coupling such as RSA, and flexible heart rate regulation.

In humans, cardiac vagal regulation reflects the capacity to shift between engagement and withdrawal in response to environmental demands. Higher baseline RSA or HF-HRV is associated with improved emotional regulation, reduced inflammation, and more adaptive stress responses, corresponding to lower cardiovascular risk [194,195].

#### 4.4.2. Social Interaction and HRV: Empirical Evidence

Positive social behaviors, including touch, empathy, shared laughter, and synchronized activities, are associated with enhanced cardiac vagal regulation. Dyadic synchrony during mother–infant bonding or romantic interaction increases HF-HRV and RSA [29]. Similarly, group activities such as singing or meditation elevate HF-HRV and reduce blood pressure, particularly in aging and clinical populations [196,197].

Neuroimaging studies support these findings, showing that activity in the medial prefrontal cortex and anterior cingulate cortex correlates with perceived social support and HRV, indicating integration between emotional processing and brainstem-mediated cardiovascular regulation [198,199].

#### 4.4.3. OT–Vagal Interactions

**Figure 9:**
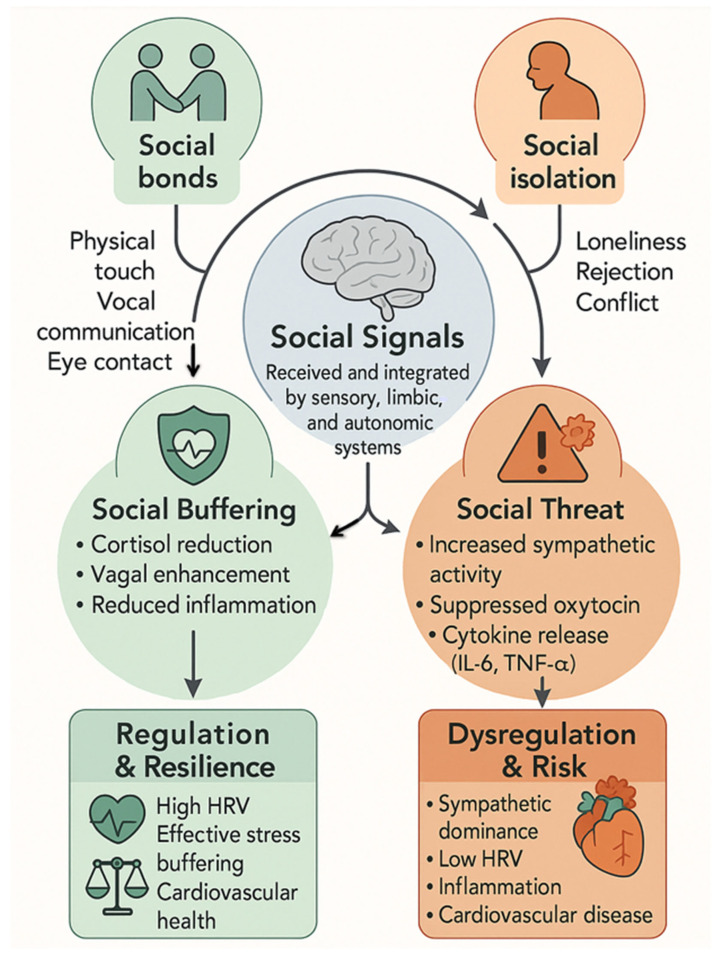
Social Signals and Physiological Regulation. Social bonds generate affiliative signals that enhance vagal activity, reduce cortisol, and lower inflammation, promoting cardiovascular resilience through high HRV and balanced autonomic function. In contrast, social isolation produces threat signals that increase sympathetic activation, suppress oxytocin (OT), and elevate cytokine release, leading to dysregulation marked by hypertension, low heart rate variability (HRV), and chronic inflammation. Together, these pathways highlight how social environments shape health via neuroendocrine–autonomic mechanisms.

Oxytocin and vagal regulation are closely linked. OT enhances cardiac vagal output indirectly through projections from the PVN to brainstem autonomic nuclei. OTRs are expressed in the NTS and DMNX, where OT modulates integration of visceral input and vagal efferent signaling. Through these mechanisms, OT influences brainstem circuits that regulate cardioinhibitory output. Rather than directly activating nucleus ambiguus motor neurons, OT modulates cardiac vagal activity through NTS processing and downstream dorsal vagal complex pathways (Figure 9).

Increased vagal afferent activity, in turn, influences hypothalamic OT release, establishing a closed-loop regulatory system in which afferent visceral signaling and oxytocinergic modulation of brainstem nuclei continuously recalibrate cardiac vagal output in response to social and environmental cues. This bidirectional loop is proposed to support social engagement, physiological flexibility, and cardiovascular stability [173,186,187].

Interventions that elevate endogenous OT, including warm social contact, caregiving interactions, or intranasal OT administration, are associated with increased HF-HRV and RSA, which are indices of vagal-mediated cardiac regulation [52,76]. This framework provides a mechanistic explanation for the cardioprotective effects of social engagement. In contrast, social isolation, chronic loneliness, or insecure attachment are associated with reduced vagal-mediated HRV, diminished OT signaling, elevated inflammation, and heightened cardiovascular reactivity [117].

## 5. Neuropeptidergic Systems: OT, VP, CRH, UCNs

### 5.1. Anatomy, Synthesis, and Receptor Distribution

OT, VP, CRH, UCNs are synthesized in discrete hypothalamic and brainstem nuclei and released centrally and peripherally to regulate autonomic, behavioral, and cardiovascular functions. Despite distinct molecular origins, these peptides form an integrated network linking stress perception with physiological homeostasis.

#### 5.1.1. OT and VP: Synthesis and Pathways

OT and VP are nonapeptides synthesized primarily in the PVN and SON nuclei of the hypothalamus. Magnocellular neurons project to the posterior pituitary, releasing OT and VP into the circulation, while parvocellular neurons project to brainstem autonomic nuclei, the spinal cord, and limbic forebrain, enabling widespread regulation of visceral and behavioral states [180,200].

OTRs, which are G-protein-coupled receptors, are widely expressed in the brain, including the amygdala, nucleus accumbens, and dorsal vagal complex, as well as in peripheral tissues such as the heart, vasculature, and gastrointestinal tract. VP acts through V1a, V1b, and V2 receptors, with V1aR and V1bR expressed in vascular smooth muscle, brain, and adrenal glands, and V2R primarily in the kidney.

These systems are sexually dimorphic and shaped by sex steroids and early-life experience, with overlapping but distinct functional roles. VP exerts stronger vasoconstrictive and ACTH-releasing effects, whereas OT is more closely associated with social bonding, vagal regulation, and cardiovascular protection [19,201].

#### 5.1.2. CRH and UCNs: Corticolimbic Integration

CRH is synthesized in the PVN, central amygdala, and BNST, where it initiates HPA axis activation by stimulating ACTH release from the anterior pituitary. It is also released within the brain to regulate fear, arousal, and sympathetic activity. CRH receptors include CRHR1 and CRHR2, which differ in distribution and function. CRHR1 predominates in limbic and cortical regions and mediates acute stress responses, whereas CRHR2 is more abundant in subcortical and autonomic centers, including the dorsal raphe, NTS, and heart, and supports recovery, vagal tone, and stress adaptation [202,203].

**Figure 10:**
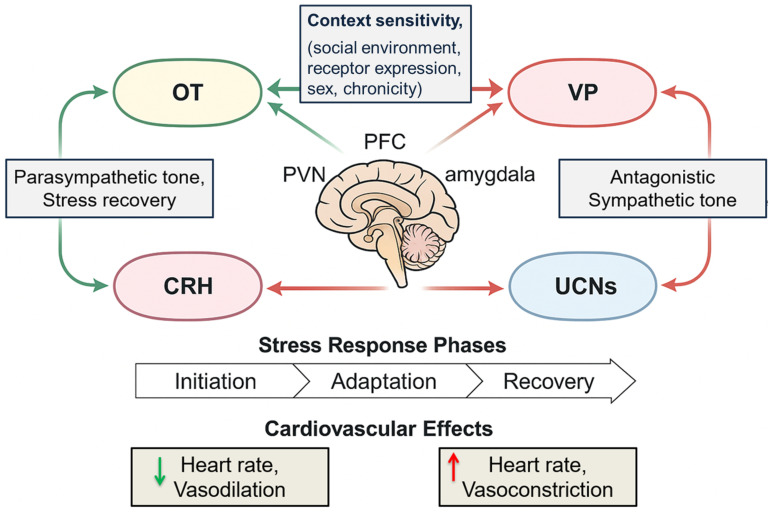
Interactions and Crosstalk Among OT, VP, CRH, and UCNs in Stress and Cardiovascular Regulation.

UCNs are CRH-related peptides with high affinity for CRHR2. UCN1 is localized to the Edinger–Westphal nucleus, while UCN2 and UCN3 are expressed in hypothalamic and brainstem regions. Peripherally, UCNs are found in the heart, vasculature, and adrenal glands, where they promote vasodilation, enhance cardiac contractility, and exert anti-inflammatory effects [204] (Figure 10).

This schematic depicts how oxytocin (OT), vasopressin (VP), corticotropin-releasing hormone (CRH), and urocortins (UCNs) interact across four domains: (1) synergistic vs. antagonistic effects, with OT/UCNs enhancing parasympathetic and anti-inflammatory responses while VP/CRH amplify sympathetic arousal; (2) temporal dynamics, where CRH/VP dominate early stress and OT/UCNs shape recovery; (3) context sensitivity, influenced by social environment, sex, chronicity, and receptor expression; and (4) cardiovascular hormesis, with peptides tuning mitochondrial, redox, vagal, and endothelial resilience. Dysregulation of this network contributes to cardiometabolic and neuropsychiatric diseases.

#### 5.1.3. Cardiovascular Relevance of Receptor Topography

The anatomical distribution of these peptides and their receptors position them to regulate both autonomic output and vascular tone. For example: OTRs in the heart and vasculature mediate nitric oxide–dependent vasodilation and cardioprotection. V1aR stimulation increases vasoconstriction and afterload. And CRHR2 activation by UCN2 and UCN3 promotes vasodilation, anti-hypertrophy, and cardiac stress resilience. Neuropeptide projections from the PVN and SON to the NTS and dorsal vagal complex allow for fine-tuned regulation of baroreflex sensitivity, HRV, and stress recovery [187]. Understanding the differential receptor profiles of these neuropeptides is crucial for developing targeted therapies, such as CRHR2 agonists, selective OTR modulators, or VP antagonists, to manage stress-induced cardiovascular disorders.

### 5.2. Behavioral and Autonomic Effects

The neuropeptidergic systems of OT, VP, CRH, and UCNs serve as key mediators between psychological states and somatic physiology. Their influence on behavior and autonomic regulation forms the neurochemical substrate by which social context and stress exposure translate into cardiovascular outcomes.

#### 5.2.1. OT: Social Affiliation and Vagal Modulation

OT promotes prosocial behavior, trust, and emotional regulation, particularly during social contact and caregiving. These effects are associated with increased parasympathetic tone, improved HRV, and reduced sympathetic activity, contributing to cardiovascular protection [18]. Central OT acts on the NTS and DMNX, enhancing baroreflex sensitivity and vagal control. In humans, intranasal OT increases RSA and reduces blood pressure during social stress [28].

#### 5.2.2. VP: Social Vigilance and Sympathetic Activation

VP exerts context-dependent effects ranging from social vigilance to aggression. It modulates hypothalamic and brainstem circuits governing arousal, blood pressure, and adrenocorticotropic hormone (ACTH) release. Through V1a receptor activation, VP increases sympathetic tone, promotes vasoconstriction, and amplifies HPA activity, particularly during social threat [15,205]. In animal models, VP antagonism reduces anxiety-like behavior and stress-induced hypertension.

#### 5.2.3. CRH and UCNs: Stress Reactivity and Recovery

CRH drives stress-induced arousal, increasing sympathetic activity, HPA axis output, and behavioral vigilance. Its central actions via CRHR1 enhance cardiovascular reactivity, while chronic activation contributes to endothelial dysfunction and inflammation [143]. In contrast, UCN2 and UCN3 act through CRHR2 to promote recovery, parasympathetic restoration, and vascular stability. These peptides counterbalance CRH effects by reducing sympathetic activity and supporting anti-arrhythmic and vasodilatory responses [206].

#### 5.2.4. Integration and Cardiovascular Relevance

Together, these neuropeptides form a dynamic balance in which OT and UCNs support social buffering and autonomic flexibility, while VP and CRH promote vigilance and allostatic effort. Dysregulation, including reduced OT signaling, excess VP activity, or persistent CRH drive, shifts this balance toward sympathetic dominance, reduced HRV, endothelial dysfunction, and cardiovascular disease [207]. Thus, neuropeptidergic regulation plays a direct role in cardiometabolic risk.

### 5.3. Interaction with HPA Axis and Cardiovascular System

OT, VP, CRH, and UCNs are closely integrated with the HPA axis, forming a network that regulates autonomic tone, cardiovascular function, and stress responses. Their interactions determine the balance between adaptive regulation and vulnerability.

#### 5.3.1. CRH and VP: Central Drivers of HPA and Sympathoexcitation

CRH initiates HPA axis activation by stimulating ACTH release from the anterior pituitary, leading to cortisol secretion. VP, particularly via V1b receptors, enhances this response under chronic stress [143,208]. Together, CRH and VP increase sympathetic output, cardiac contractility, vascular tone, and blood pressure while reducing vagal activity. Sustained activation contributes to hypertension, endothelial dysfunction, and impaired baroreflex sensitivity [209].

#### 5.3.2. OT and UCNs: Modulators of HPA–Cardiovascular Reactivity

In contrast, OT and UCNs exert buffering effects. OT inhibits excessive HPA activation through feedback on limbic and hypothalamic circuits, reducing ACTH and cortisol responses while enhancing parasympathetic tone and vascular relaxation [180,210,211]. UCN2 and UCN3 activate CRHR2 receptors in the heart, vasculature, and brainstem, promoting vasodilation, improving cardiac function, and counteracting CRH-mediated effects. These peptides play key cardioprotective roles, particularly in ischemia–reperfusion injury and heart failure.

### 5.4. Role in Resilience and Social Adaptation

OT, VP, CRH, and UCNs form a coordinated system that supports resilience and social adaptation. OT is central to affiliative behavior and social buffering, reducing amygdala reactivity, modulating HPA activity, and increasing vagal tone [161,180]. These effects improve emotional regulation, HRV, and stress responses, with higher endogenous OT associated with better coping following adversity [18,212].

VP contributes to social vigilance and memory for social cues. Although it can amplify stress responses via V1a signaling, it may support adaptive social behavior in stable environments [200,213].

CRH and UCNs regulate both stress activation and recovery. CRH promotes alertness but contributes to anxiety and dysregulation when sustained. In contrast, UCN2 and UCN3 activate CRHR2 to produce anxiolytic, anti-inflammatory, and cardioprotective effects, supporting recovery and resilience [214].

These systems interact extensively. OT and VP share overlapping projections, while CRH and UCNs modulate their activity depending on context, sex, and receptor distribution [210,215]. Together, they form an integrated network that enables flexible responses to both threat and social connection.

In supportive environments, this network enhances parasympathetic tone, reduces inflammation, and stabilizes cardiovascular function. In contrast, chronic stress or social isolation disrupts this balance, increasing allostatic load and vulnerability to psychiatric and cardiovascular disorders [45]. The timing and coordination of neuropeptidergic responses, rather than their absolute levels, determine whether stress leads to resilience or disease.

This network represents a molecular interface linking social context, psychological state, and cardiovascular function. Its role in resilience underscores a broader principle: adaptation is not solely individual but emerges from socially embedded neuroendocrine regulation. Understanding and targeting this system offers important opportunities to improve psychological and cardiovascular health.

## 6. Brain–Heart Communication via Autonomic and Peptidergic Pathways

### 6.1. Central Autonomic Network: PVN, NTS, Amygdala, PFC

Cardiovascular regulation is centrally coordinated by the central autonomic network (CAN), a distributed system integrating visceral, emotional, cognitive, and endocrine signals to modulate autonomic output and neuropeptidergic tone [216,217]. Key components include the PVN, NTS, amygdala, and prefrontal cortex (PFC).

#### 6.1.1. Paraventricular Nucleus

The PVN is a major regulator of autonomic and neuroendocrine function. It contains neurons that release OT, VP, and CRH into the pituitary and descending autonomic pathways. Integrating inputs from limbic and brainstem regions, it coordinates sympathetic and parasympathetic activity. During stress, PVN activation modulates HPA output and vagal tone, influencing heart rate and blood pressure [187,209].

#### 6.1.2. Nucleus Tractus Solitarius

The NTS is the primary brainstem relay for baroreceptor and chemoreceptor input via cranial nerves IX and X. It projects to autonomic centers including the DMNX and rostral ventrolateral medulla (RVLM), and sends glutamatergic projections to the PVN, enabling integration of visceral signals and reflexive autonomic regulation [218,219]. OT and UCN signaling within the NTS enhances baroreflex sensitivity and buffers sympathetic activation [220,221].

#### 6.1.3. Amygdala

The amygdala processes emotional salience and threat, influencing autonomic output through projections to hypothalamic and brainstem centers. Chronic stress sensitizes amygdala activity, increasing sympathetic tone, reducing HRV, and sustaining cardiovascular arousal [132]. CRH and VP further modulate amygdala excitability, linking stress to long-term cardiovascular risk [128].

#### 6.1.4. Prefrontal Cortex (PFC)

The medial and ventrolateral PFC provide top-down regulation of autonomic function via inhibitory projections to the amygdala and hypothalamus. PFC integrity is associated with higher HRV, effective emotional regulation, and resilience to stress [47]. Dense OT receptor expression in the PFC supports vagal activation and suppression of sympathetic output, facilitating social engagement and stress regulation [18,180].

#### 6.1.5. Integrated Role in Brain–Heart Regulation

These regions form a hierarchical loop integrating cognitive appraisal (PFC), emotional processing (amygdala), visceral sensing (NTS), and neuroendocrine coordination (PVN). Disruption at any level, due to chronic stress, trauma, or inflammation, can lead to dysautonomia and increased cardiovascular risk [222]. Interventions such as mindfulness, vagal stimulation, pharmacologic modulation, and social buffering may restore this regulatory balance.

### 6.2. Neurocardiac Feedback Loops and HRV

The heart functions within a bidirectional brain–heart axis, where afferent and efferent signaling modulates physiological and emotional states. HRV is a key biomarker of this system.

#### 6.2.1. HRV as a Window into Neurovisceral Integration

HRV reflects beat-to-beat variability in heart rate, primarily governed by vagal activity. High HRV indicates flexible autonomic regulation and cardiovascular resilience, whereas low HRV reflects sympathetic dominance and increased disease risk [223,224]. It serves as an index of CAN output, particularly the balance between prefrontal regulation and amygdala-driven reactivity [225].

#### 6.2.2. Afferent and Efferent Pathways in Heart–Brain Communication

Neurocardiac feedback involves two primary pathways: afferent signals from the heart to the brain via vagal fibers projecting to the NTS, conveying information about pressure and metabolic status, and efferent autonomic signals from CAN regions, including the PVN and PFC, regulating heart rate, contractility, and vascular tone. These pathways support cardiovascular regulation, interoception, emotional processing, and social behavior [226,227].

#### 6.2.3. Stress, HRV, and Feedback Disruption

Chronic stress and trauma impair neurocardiac feedback by reducing vagal tone, increasing sympathetic activity, and diminishing baroreflex sensitivity. These changes lower HRV and are associated with poorer cardiovascular outcomes and increased psychological distress [45]. Interventions such as mindfulness, biofeedback, vagal stimulation, and oxytocinergic modulation can restore HRV and improve neurovisceral integration [186,224].

#### 6.2.4. Hormetic Modulation of HRV

Hormetic stressors, including exercise, thermal exposure, and intermittent fasting, can enhance HRV through central and peripheral adaptations. These stimuli increase parasympathetic activity, improve baroreflex sensitivity, and engage brainstem–hypothalamic circuits involved in adaptive regulation [72]. OT and CRH receptor signaling within the NTS and PVN also modulate autonomic responses and help preserve HRV during stress [220,221].

### 6.3. OT, VP, and CRH–UCN Modulation of Cardiac Function

OT, VP, CRH, UCNs regulate cardiovascular function through both central autonomic pathways and direct peripheral effects on cardiac tissue, endothelium, and systemic hemodynamics. These peptides influence cardiac output, rhythm stability, and vascular tone under basal and stress conditions, representing key components of the brain–heart axis.

#### 6.3.1. OT: Cardiac Protection and Vagal Facilitation

OT promotes parasympathetic dominance, enhancing vagal tone, baroreflex sensitivity, and resistance to sympathetic overactivation. OTRs are expressed in cardiomyocytes, endothelial cells, and autonomic centers including the NTS and PVN. OT increases HRV and improves cardiac function in ischemia–reperfusion and heart failure models [220,221], largely through nitric oxide signaling, improved perfusion, and anti-apoptotic mechanisms.

#### 6.3.2. VP: Bidirectional Effects on Cardiovascular Stability

VP exerts dose-dependent effects on cardiovascular function. At physiological levels, it supports vascular tone and blood pressure during hypovolemia and orthostatic stress, while centrally modulating baroreflex integration [228,229]. In chronic stress or heart failure, however, elevated VP promotes vasoconstriction, fluid retention, and cardiac remodeling. Increased VP is associated with reduced HRV, higher afterload, and worse outcomes in HFpEF and arrhythmias [49], supporting the use of V1a/V2 antagonists in therapy.

#### 6.3.3. CRH and UCNs: Stress Peptides with Cardioprotective Roles

CRH initiates HPA activation and sympathetic arousal, whereas UCNs primarily mediate peripheral cardiovascular effects through CRHR2. CRHR2 activation promotes vasodilation, reduces afterload, and limits inflammation [230]. UCN2 and UCN3 enhance myocardial contractility without increasing oxygen demand and protect against ischemic injury. Early clinical studies suggest benefits in stress cardiomyopathy, HFpEF, and endothelial dysfunction, supporting interest in CRHR2-targeted therapies [204,231].

#### 6.3.4. Integration in Cardiovascular Homeostasis

The balance among OT, VP, CRH, and UCNs coordinates autonomic, endocrine, and local cardiac responses to stress. Disruption of this network, due to chronic stress, trauma, or metabolic dysregulation, shifts the system toward sympathetic dominance, inflammation, and reduced cardiac resilience. Understanding this integration is essential for developing unified models of neurovisceral regulation.

#### 6.3.5. Apelin and Elabela as Peripheral Mediators of Cardiovascular Hormesis

Apelin and Elabela (ELA) represent key peripheral mediators of cardiovascular hormesis. Through activation of the APJ receptor in endothelial cells, cardiomyocytes, and renal tissue, they engage PI3K–Akt, eNOS/NO, and ERK1/2 pathways that support adaptive responses [232,233]. Mild stressors such as exercise or intermittent ischemia increase apelinergic signaling, enhancing mitochondrial efficiency, nitric oxide bioavailability, and resistance to oxidative injury while limiting apoptosis and fibrosis [234].

These peptides mimic preconditioning effects by reducing infarct size, improving ventricular recovery, and restoring endothelial function [235]. ELA levels decline with increasing disease severity, suggesting loss of peripheral adaptive capacity, whereas Apelin may increase compensatorily in heart failure [232]. Together, they form a vasodilatory, anti-inflammatory axis that complements central OT–VP–CRH–UCN signaling by translating stress exposure into adaptive cardiovascular responses.

### 6.4. Dynamic Coupling of Emotion and Cardiovascular States

Emotions are embodied processes that engage autonomic, vascular, and visceral systems in real time. States such as fear, attachment, anger, and grief produce measurable changes in heart rate, blood pressure, and vascular tone through central–peripheral integration, shaping cardiovascular resilience and disease risk [227,236].

#### 6.4.1. Neurocardiac Integration of Emotion

Emotionally salient stimuli activate the amygdala, insula, medial prefrontal cortex (mPFC), anterior cingulate cortex (ACC), and hypothalamus, which project to brainstem autonomic centers including the NTS and RVLM. These circuits regulate heart rate and vascular tone through coordinated sympathetic and vagal output, enabling adaptive behavioral responses.

#### 6.4.2. Cardiac Interoception and Emotional Processing

Cardiac signals influence emotional experience via interoceptive pathways. Baroreceptor and vagal afferents transmit cardiovascular information to the NTS and onward to insula–ACC–mPFC networks involved in emotional awareness [237,238]. Baroreceptor activation can dampen threat responses by modulating amygdala activity. Individuals with higher HRV exhibit improved emotional regulation and reduced cardiovascular reactivity [239].

#### 6.4.3. Neuropeptides as Emotional–Cardiac Couplers

Neuropeptides such as OT, VP, CRH, and UCNs link emotional states to cardiovascular responses. OT enhances social bonding while promoting vagal dominance and reducing cardiovascular reactivity [220,221]. CRH and UCNs modulate arousal and cardiovascular output during stress [156]. Together, these systems integrate emotional salience, autonomic regulation, and cardiac function.

#### 6.4.4. Emotional Rigidity and Cardiovascular Risk

Disruption of emotional–cardiac coupling, whether blunted or hyperactive, increases vulnerability to cardiovascular and psychiatric disorders. Reduced HRV and sustained sympathetic activation are associated with depression, anxiety, PTSD, and arrhythmias. Chronic activation of these circuits leads to baroreflex impairment, endothelial dysfunction, and structural cardiac changes [240,241]. Targeting neurovisceral pathways through interventions such as HRV biofeedback, social engagement, and OT-based therapies may improve both emotional and cardiovascular outcomes.

### 6.5. Influence of Neuropeptides on Vagal Tone and Inflammation

The vagus nerve is a key conduit for brain–heart communication, regulating both cardiac function and immune responses. Neuropeptides such as OT, VP, CRH, UCNs modulate vagal output and systemic inflammation, contributing to cardiovascular homeostasis and resilience.

#### 6.5.1. Neuropeptidergic Enhancement of Vagal Tone

Vagal tone, indexed by HF-HRV and baroreflex sensitivity, reflects parasympathetic adaptability. OT enhances vagal efferent activity via projections from the PVN to the dorsal vagal complex and nucleus ambiguus. Experimental and clinical studies show that OT increases the deceleration of the vagal-mediated heart rate, promotes relaxation, and improves autonomic stability [220,221].

VP also contributes by modulating baroreflex gain and supporting parasympathetic restraint of cardiac excitability. UCN2 and UCN3, acting through CRHR2, exert cardiodepressive and vasodilatory effects partly by enhancing vagal influence on the sinoatrial node and reducing sympathetic tone [221,242].

#### 6.5.2. Vagal–Immune Reflex and Neuropeptidergic Modulation

The cholinergic anti-inflammatory pathway (CAP) is a vagus-dependent circuit in which acetylcholine acts on α7 nicotinic acetylcholine receptors on immune cells to suppress pro-inflammatory signaling, including TNF-α, IL-1β, and NF-κB [243,244].

Neuropeptides enhance this pathway by increasing vagal tone and directly modulating immune function. OT inhibits NF-κB activation, reduces cytokine expression, and promotes regulatory T-cell activity, partly through vagal mechanisms [173]. UCNs also exhibit anti-inflammatory effects in models of sepsis, ischemia–reperfusion, and vascular injury, highlighting their role in linking autonomic regulation with immune control [245].

#### 6.5.3. Clinical Implications: Inflammation and Cardiovascular Risk

Reduced vagal tone and elevated inflammation are central features of cardiometabolic disease, heart failure, and stress-related cardiovascular conditions. Dysregulation of the OT–VP–CRH–UCN network contributes to this imbalance by weakening vagal signaling and promoting sympathetic dominance.

Individuals with depression, PTSD, or chronic social isolation often exhibit reduced HRV, elevated C-reactive protein (CRP), and increased endothelial activation, linking affective states to systemic inflammation and cardiovascular risk [48]. These findings support the potential of neuropeptide-targeted interventions, including pharmacologic approaches such as OT analogs and CRHR2 agonists, as well as behavioral strategies such as social engagement and vagal biofeedback, to restore autonomic balance, reduce inflammation, and improve cardiovascular outcomes.

## 7. Interactions and Crosstalk Among OT, VP, CRH, and UCNS

### 7.1. Synergistic and Antagonistic Roles

OT, VP, CRH, UCNs form an integrated network with both synergistic and antagonistic interactions that shape responses to stress, social context, and cardiovascular demand. Although each peptide has distinct receptors and pathways, they converge on shared autonomic, endocrine, and inflammatory targets, making their balance critical for homeostasis and disease risk. These synergistic and antagonistic relationships are summarized in Figure 10.

#### 7.1.1. Synergistic Interactions

OT and UCNs often act synergistically to promote parasympathetic tone, stress recovery, and cardiovascular resilience. Both attenuate HPA axis activation, enhance vagal-mediated bradycardia and baroreflex sensitivity, and reduce inflammatory signaling. OT and UCN2, for example, support CRHR2-mediated vasodilation, reduce sympathetic excitability, and protect against endothelial dysfunction [105,245]. In social contexts, OT and VP may also cooperate to support attachment and social memory, with VP contributing to vigilance and contextual awareness under mild stress [181,212].

#### 7.1.2. Antagonistic Interactions

OT and VP frequently exert opposing effects. VP promotes arousal, vasoconstriction, and sympathetic activation via V1a receptors, whereas OT enhances vagal tone, social bonding, and cardiovascular stability. Elevated VP is associated with hypertension, anxiety, and reduced HRV, contrasting OT’s buffering role [58].

CRH and VP can also drive maladaptive responses under chronic stress. While VP enhances CRH-mediated ACTH release acutely, sustained activation leads to HPA overdrive, glucocorticoid toxicity, and autonomic dysregulation [136]. UCNs add complexity by counteracting CRH effects. UCN2 and UCN3, acting through CRHR2, reduce sympathetic output and promote vasodilation, whereas CRH acting via CRHR1 increases arousal and blood pressure. The balance between CRHR1 and CRHR2 signaling determines whether outcomes are adaptive or pathological [59].

#### 7.1.3. Context-Dependent Outcomes

The net effect of OT–VP–CRH–UCN interactions depends on receptor distribution, tissue specificity, timing, and individual factors such as sex, developmental history, and social environment. In supportive contexts, OT-mediated signaling predominates, buffering stress responses. Under threat or isolation, VP and CRH signaling dominate, increasing sympathetic activation and vascular strain. These dynamics have therapeutic implications. Targeted strategies, such as CRHR2 agonists or OTR-enhancing approaches, may harness beneficial interactions while limiting maladaptive signaling.

### 7.2. Temporal and Context-Specific Peptide Responses

The release and effects of these peptides are dynamic, temporally structured, and context-sensitive, shaping cardiovascular and behavioral outcomes across conditions.

#### 7.2.1. Temporal Dynamics of Peptidergic Release

OT and VP are released in phasic bursts in response to stimuli such as social interaction, exercise, or stress, producing rapid yet sustained modulatory effects [246]. Acute exercise, for instance, increases both OT and cortisol, supporting vagal tone and metabolic readiness.

CRH and VP dominate early stress responses, initiating HPA activation and sympathetic arousal. Prolonged activation contributes to dysregulation and vascular pathology [136]. In contrast, UCN2 and UCN3 are more prominent in later phases, promoting recovery, parasympathetic restoration, and feedback inhibition of HPA activity [147,247]. This pattern reflects a temporal sequence in which CRH–VP drive initial arousal, followed by OT–UCN-mediated recovery and stabilization.

#### 7.2.2. Context-Dependent Expression and Function

Peptide responses also vary with social and environmental context. In affiliative settings, OT predominates, supporting reduced blood pressure, enhanced HRV, and safety signaling. Under threat or isolation, VP and CRH dominate, increasing sympathetic tone and vascular resistance [59].

**Figure 11:**
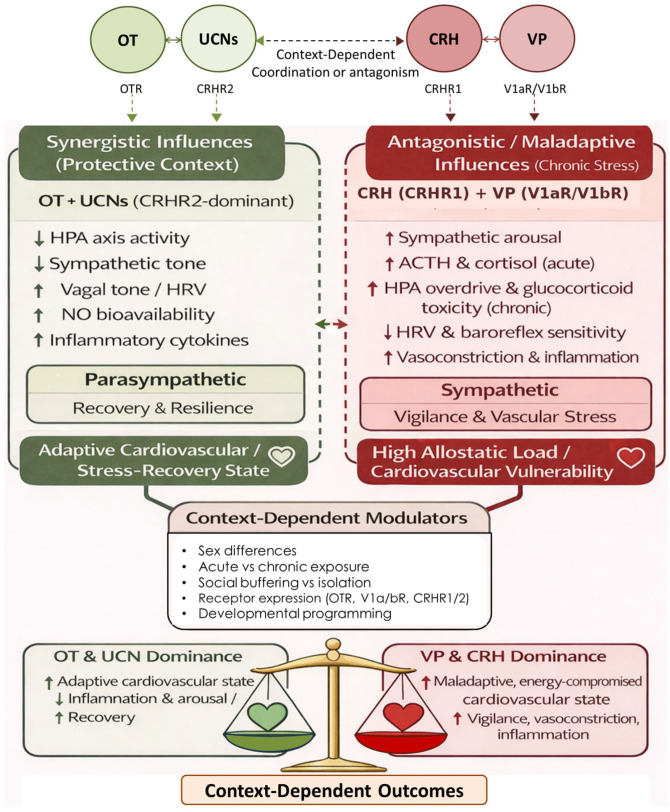
Interactions and crosstalk among OT, VP, CRH, and UCNs in stress and cardiovascular regulation. Oxytocin (OT), vasopressin (VP), corticotropin-releasing hormone (CRH), and urocortins (UCNs) constitute an integrated neuropeptidergic network that regulates autonomic tone, hypothalamic–pituitary–adrenal (HPA) axis activity, vascular function, and inflammatory signaling. In protective or recovery-oriented contexts, OT and UCNs, acting primarily through oxytocin receptors (OTR) and corticotropin-releasing hormone receptor 2 (CRHR2), synergistically suppress HPA axis activity, reduce sympathetic tone, enhance vagal activity and heart rate variability (HRV), increase nitric oxide bioavailability, and limit inflammatory cytokine production, thereby supporting cardiovascular resilience and stress recovery. During chronic or socially unbuffered stress, CRH signaling through CRHR1 together with VP signaling via V1aR/V1bR promotes sympathetic arousal, acute ACTH and cortisol release, and, when sustained, HPA axis overdrive, glucocorticoid toxicity, reduced HRV and baroreflex sensitivity, vasoconstriction, and inflammation. The balance between OT–UCN and CRH–VP signaling is context-dependent and shaped by factors such as sex, stress duration, social environment, receptor expression, and developmental programming. Shifts toward CRH–VP dominance increase allostatic load and cardiovascular vulnerability, whereas OT–UCN dominance favors adaptive cardiovascular function and efficient recovery from stress.

Individual variability further modulates these responses. Sex differences, early life adversity, circadian rhythms, and genetic polymorphisms in receptors such as OXTR, AVPR1a, and CRHR2 influence baseline activity and stress reactivity. Individuals with low social support or prior trauma often exhibit heightened VP and CRH responses and reduced OT feedback, contributing to autonomic rigidity and cardiovascular vulnerability [138,248]. These context-dependent shifts in peptide dominance and receptor-mediated signaling are illustrated in Figure 11.

#### 7.2.3. Implications for Stress Adaptation and Therapeutic Timing

The temporal and contextual dynamics of these peptides have important therapeutic implications. Interventions should consider both timing and environment:

- Oxytocin-based interventions may be most effective during recovery or social engagement phases.
- CRHR2 agonists or UCN analogs may better target maladaptive HPA activation following prolonged stress.
- Peptide-based therapies should be tailored to individual physiological state and psychosocial context.

Together, the coordinated, time-dependent activity of OT, VP, CRH, and UCNs forms a dynamic regulatory framework in which molecular signaling is shaped by both internal physiology and external social environment.

### 7.3. Integration in Stress Response Phases

OT, VP, CRH, and UCNs do not operate as isolated mediators. Instead, their dynamic integration across different phases of the stress response, including initiation, adaptation, and recovery, enables a more flexible and finely tuned system of neurovisceral regulation. This phase-dependent orchestration allows the organism to mount an immediate defense while preparing for recovery and long-term resilience. These phase-dependent interactions are illustrated in Figure 10.

#### 7.3.1. Initiation Phase: Alarm and Mobilization

At threat onset, CRH neurons in the PVN activate the HPA axis, driving ACTH and cortisol release. VP potentiates this response via V1b receptors, amplifying arousal, vigilance, and cardiovascular activation [136]. During this phase, OT signaling is generally reduced, reflecting a shift away from affiliative and parasympathetic processes, although social input may partially counteract this effect. CRH and UCN1 activity in the amygdala and bed nucleus of the stria terminalis further promote defensive behaviors.

#### 7.3.2. Adaptation Phase: Sustained Regulation

With persistent stress, the system transitions to a regulated state. UCN2 and UCN3, acting through CRHR2, counterbalance CRH-driven activation in limbic and brainstem regions, reducing excessive arousal and promoting adaptive coping [249,250]. OT levels begin to recover, particularly under safe or socially supportive conditions, facilitating vagal rebound, blood pressure normalization, and restoration of prefrontal control. OT enhances parasympathetic tone and reduces cortisol output, supporting prosocial coping. VP effects become context-dependent, with central actions maintaining arousal while peripheral signaling supports vascular tone and fluid balance [58].

#### 7.3.3. Recovery Phase: Termination and Repair

Resolution of stress initiates a recovery phase marked by reduced CRH–VP activity and engagement of reparative processes. UCNs, particularly UCN3 via CRHR2, reduce sympathetic tone and support myocardial recovery, perfusion, and redox balance [251]. OT contributes to emotional processing, immune regulation, and anti-inflammatory signaling, promoting autonomic recalibration across systems.

Together, OT and UCNs act as regulatory brakes, limiting prolonged HPA activation, preserving endothelial function, and restoring social engagement, a key component of sociostasis [211].

#### 7.3.4. Maladaptive Transitions Between Phases

Disruptions in phase transitions, such as prolonged CRH–VP activation or inadequate OT–UCN recovery, impair adaptive capacity. Chronic stress reduces CRHR2 and OTR sensitivity, diminishes vagal tone, and increases risk for anxiety, cardiovascular dysfunction, and stress-induced cardiomyopathy [48]. Understanding these temporal dynamics is essential for restoring adaptive stress responses.

### 7.4. Influence of OT, VP, CRH, and UCNs on Cardiovascular Hormesis

Cardiovascular hormesis reflects a biphasic response in which mild stress promotes adaptation, while excessive exposure leads to dysfunction. OT, VP, CRH, and UCNs shape this response by modulating redox balance, mitochondrial function, autonomic tone, and vascular plasticity.

OT enhances resilience by increasing vagal tone, reducing sympathetic activity, and promoting nitric oxide production. It improves mitochondrial efficiency and reduces oxidative stress, thereby supporting the adaptive window. In experimental models, OT improves post-ischemic recovery through Akt and MAPK signaling [95,252].

VP exhibits context-dependent effects. At low levels, it supports vascular tone and perfusion during transient stress, whereas chronic elevation impairs baroreflex sensitivity and promotes endothelial dysfunction, reflecting loss of hormetic balance [253].

CRH and UCNs, particularly via CRHR2, exert cardioprotective and anti-inflammatory effects at physiological levels. UCN2 and UCN3 improve cardiac output, reduce vascular resistance, and buffer sympathetic activation [206]. These effects are dose-dependent, highlighting their role in defining hormetic thresholds.

These peptides also influence the duration of adaptive responses. OT and UCNs can shorten recovery time and extend protective phases following transient stress. Their interaction enhances anti-apoptotic and antioxidant signaling, particularly in aging or metabolically compromised states [251].

Integration of neuropeptide signaling into cardiovascular hormesis provides a framework linking stress history, emotional state, and cardiovascular outcomes. It also explains variability in adaptive capacity based on peptide tone, receptor expression, and prior exposure (Figure 11).

## 8. Dysregulation and Disease States: When Systems Fail

### 8.1. Hypo- or Hyperactivation of OT, VP, CRH Systems

Balanced activity of OT, VP, and CRH is essential for cardiovascular and emotional homeostasis. Dysregulation, whether hypo- or hyperactivation, promotes maladaptive states, particularly under chronic stress, trauma, or social deprivation.

Hypoactivation of OT is commonly observed following early life adversity or persistent isolation and is associated with reduced vagal tone, impaired baroreflex sensitivity, and diminished resilience to cardiovascular stress [211]. Behaviorally, low OT levels correlate with reduced social engagement, anhedonia, and increased inflammation and oxidative stress, all contributing to cardiometabolic risk.

In contrast, chronic VP hyperactivation increases vascular tone, sodium retention, and sympathetic drive. Elevated VP is linked to endothelial dysfunction, hypertension, HFpEF, and increased arrhythmic risk [49]. CRH hypersecretion, characteristic of prolonged stress and psychiatric disorders, sustains HPA overactivity, glucocorticoid excess, inflammation, and autonomic imbalance, contributing to endothelial injury, cardiac remodeling, and reduced HRV [136].

Context-specific hypoactivation of CRH or UCN pathways has also been observed in metabolic and cardiovascular disorders, reflecting nonlinear breakdowns in neuropeptidergic regulation. When multiple systems fail simultaneously, such as reduced OT and UCN signaling alongside elevated VP and CRH, a “peptidergic collapse” emerges, representing a combined signature of social disconnection and cardiovascular vulnerability [179,254]. Understanding these context- and dose-dependent dynamics is critical for developing targeted interventions.

### 8.2. Chronic Inflammation and Endothelial Dysfunction

Chronic inflammation both drives and results from neuropeptidergic dysregulation, with major effects on endothelial function. The endothelium acts as a dynamic interface integrating neurohormonal, immune, and mechanical signals. Under persistent stress, endothelial function becomes impaired, characterized by reduced nitric oxide availability, impaired vasodilation, increased permeability, and enhanced leukocyte adhesion [78]. Autonomic imbalance, particularly reduced vagal tone and increased sympathetic activity, amplifies inflammation by elevating catecholamines and reducing anti-inflammatory neuropeptides such as OT and UCNs [255,256]. Elevated VP and CRH further exacerbate vasoconstriction, sodium retention, and cytokine release.

Microvascular rarefaction and endothelial senescence impair tissue perfusion and promote progression from functional to structural cardiovascular disease. Disruption of protective pathways, including OT–nitric oxide coupling and UCN-mediated endothelial regulation, accelerates vascular stiffening and atherosclerosis [179,257]. Inflammatory markers such as IL-6, TNF-α, and CRP correlate with reduced HRV, indicating concurrent impairment of autonomic and vascular function [258,259].

These processes form a self-reinforcing cycle in which neuropeptidergic dysregulation promotes inflammation, which further damages endothelial and autonomic systems, eroding cardiovascular resilience. Breaking this cycle requires integrated strategies targeting both neuroendocrine and inflammatory pathways, consistent with emerging “sociovascular” frameworks.

### 8.3. Stress Cardiomyopathy, Hypertension, and Heart Failure

Dysregulation of OT, VP, CRH, and UCN systems increases vulnerability to stress-related cardiovascular diseases, including Takotsubo cardiomyopathy, hypertension, and HFpEF. Each reflects a breakdown in neuropeptidergic modulation of autonomic, vascular, and inflammatory tone [260,261].

Takotsubo syndrome is characterized by transient left ventricular dysfunction triggered by acute stress. Impaired OT signaling and excessive CRH activity may weaken vagal buffering and amplify stress responses, contributing to myocardial stunning [48].

In hypertension, sustained sympathetic activation and reduced vasodilatory capacity drive vascular remodeling. Elevated VP and CRH reinforce vasoconstriction, sodium retention, and inflammation, while reduced OT signaling impairs nitric oxide–mediated vascular protection [50].

HFpEF involves diastolic dysfunction, systemic inflammation, and autonomic imbalance. Patients exhibit reduced HRV, impaired baroreflex sensitivity, and elevated stress mediators. Deficits in OT and UCN signaling may impair mitochondrial function and endothelial repair, further contributing to disease progression [49]. Chronic social stress and isolation, key contributors to allostatic load, are increasingly recognized as independent risk factors for HFpEF [262].

Collectively, these conditions reflect failure of neuroendocrine buffering systems that normally integrate social, emotional, and physiological signals to maintain cardiovascular flexibility. Loss of sociostasis and hormesis exposes the cardiovascular system to sustained stress, accelerating disease progression.

### 8.4. Loss of Social Buffering and Peptide Plasticity

Social buffering plays a central role in cardiovascular and systemic health by modulating neuropeptides such as OT, VP, and CRH which regulate autonomic tone, inflammation, and endocrine stress responses. When social support is absent or impaired, this buffering system collapses, leading to neuroendocrine dysregulation, autonomic rigidity, and cardiovascular dysfunction [263,264]. OT is a key mediator of social buffering. Its release, triggered by physical contact and emotional attunement, promotes parasympathetic activation, reduces cortisol, and suppresses sympathetic output [142]. Under conditions of isolation, bereavement, or caregiving burden, OT signaling becomes blunted. Reduced receptor sensitivity or genetic variation in OT pathways further weakens stress-buffering capacity, increasing HPA axis activation and cardiovascular risk [136].

Loss of social buffering also disrupts peptide plasticity, the ability of OT–VP–CRH–UCN systems to adapt to environmental demands. Chronic social stress shifts this balance toward VP and CRH dominance, promoting vasoconstriction, vigilance, and inflammatory priming. While adaptive acutely, sustained activation leads to baroreflex impairment, reduced HRV, and vascular dysfunction. UCN signaling, which normally counterbalances CRH effects, may become insufficient or uncoupled under prolonged stress [265,266].

Behaviorally, reduced social connection limits opportunities for co-regulation and recovery, diminishing neurochemical processes such as OT release and vagal activation. This accelerates allostatic load and contributes to autonomic and vascular rigidity. Clinically, chronic loneliness, trauma, and caregiving stress are associated with increased risk of hypertension, arrhythmias, and metabolic disease, even after controlling for traditional risk factors [20,243,267]. These findings highlight social integration as a core regulatory axis in cardiovascular health.

### 8.5. Syndromes of Collapse: Takotsubo, HFpEF, and Metabolic Stress Disorders

Persistent dysregulation of neuroendocrine, autonomic, and social systems can lead to a state of physiological collapse characterized by impaired adaptability, reduced recovery capacity, and heightened disease vulnerability. This state is reflected in conditions such as Takotsubo cardiomyopathy, HFpEF, and metabolic stress disorders including insulin resistance and visceral obesity [268]. These syndromes represent failures of integrated adaptive systems rather than isolated cardiovascular abnormalities.

#### 8.5.1. Takotsubo Cardiomyopathy: The Heart’s Social Breakdown

Takotsubo syndrome (TTS), often triggered by acute emotional or physical stress, exemplifies neurovisceral collapse. It mimics acute coronary syndrome but occurs without obstructive coronary disease, reflecting an extreme sympathetic surge. Elevated catecholamines, impaired baroreflex buffering, and reduced vagal tone lead to transient left ventricular dysfunction, arrhythmias, and myocardial stunning [269,270].

**Figure 12:**
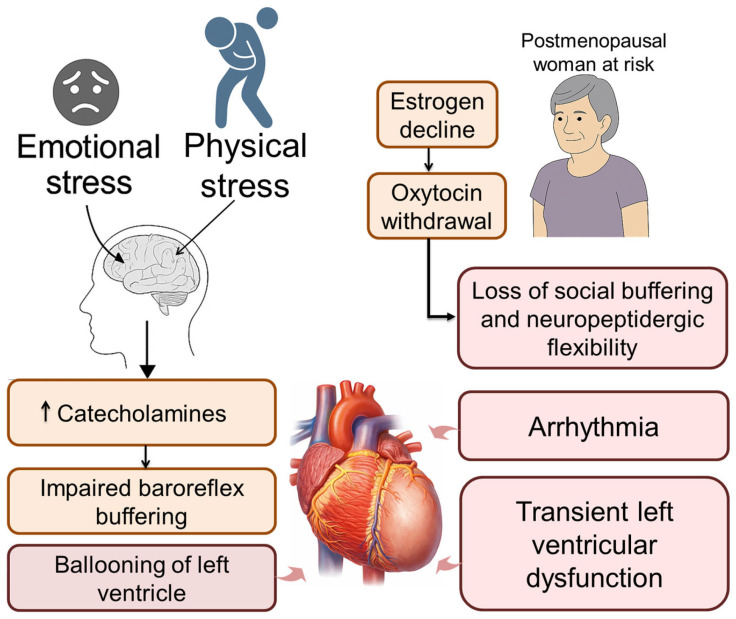
Mechanistic overview of Takotsubo Syndrome (TTS) as a neurovisceral collapse under stress. Acute emotional or physical stressors activate the hypothalamic–pituitary–adrenal (HPA) axis and sympathetic nervous system, leading to catecholamine surges and impaired baroreflex buffering. Excess catecholamines induce myocardial toxicity, resulting in transient left ventricular apical ballooning and arrhythmias characteristic of TTS. In postmenopausal women, estrogen decline and OT withdrawal reduce vagal tone and social buffering capacity, compounding vulnerability. The interaction among hormonal depletion, autonomic imbalance, and loss of neuropeptidergic flexibility constitutes the mechanistic framework for stress-induced cardiomyopathy.

The condition is more prevalent in postmenopausal women, suggesting a link between estrogen decline, reduced OT signaling, and diminished autonomic flexibility [270,271]. Patients typically exhibit reduced parasympathetic activity and impaired myocardial perfusion despite normal coronary arteries (Figure 12).

#### 8.5.2. HFpEF: The Cardiovascular Signature of Chronic Allostatic Load

Unlike the abrupt onset of Takotsubo syndrome, HFpEF develops gradually as a consequence of chronic stress exposure. It is characterized by diastolic dysfunction, vascular stiffness, and autonomic imbalance, arising from persistent low-grade inflammation, endothelial dysfunction, and reduced parasympathetic tone [272,273].

Neurohormonal dysregulation, particularly sustained CRH and VP activation, contributes to loss of vascular compliance and impaired cardiac relaxation. Elevated sympathetic activity and reduced HRV are consistently observed and correlate with diminished oxytocinergic signaling and reduced resilience to physiological stressors [274,275].

#### 8.5.3. Metabolic Stress Disorders: Beyond Energy Imbalance

Metabolic disorders, including insulin resistance, visceral adiposity, and non-alcoholic fatty liver disease, represent another axis of stress-related collapse. Chronic activation of glucocorticoid and VP pathways disrupts adipose signaling, impairs insulin sensitivity, and promotes central fat accumulation [276]. Protective systems, including OT and UCN signaling, are often suppressed under chronic social and environmental stress, exacerbating metabolic dysfunction and promoting physiological rigidity. These patterns are especially evident in individuals exposed to persistent psychosocial stress or trauma, where metabolic dysregulation co-occurs with reduced HRV, impaired vascular function, and diminished resilience [179,277].

**Figure 13:**
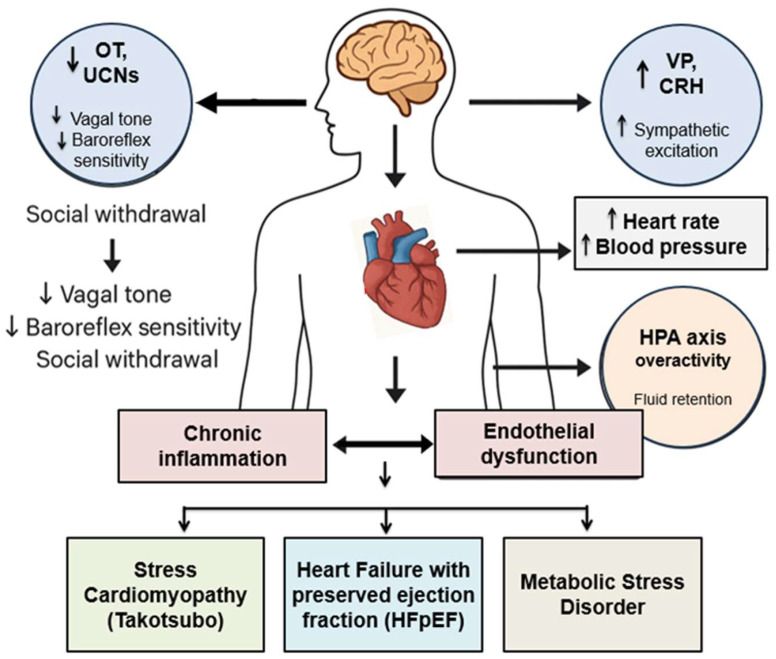
Dysregulation and Disease States: When Systems Fail.

Collectively, these conditions share common features including neuropeptidergic imbalance, autonomic dysregulation, endothelial dysfunction, and loss of protective hormetic and social feedback mechanisms. They represent syndromes of collapse in which adaptive flexibility is lost and coordination between brain, heart, and social context breaks down (Figure 13).

This schematic depicts how imbalances in Oxytocin (OT), Vasopressin (VP), and corticotropin-releasing hormone (CRH) systems drive maladaptive cardiovascular outcomes. OT hypoactivation reduces vagal tone and social buffering, fostering inflammation and stress vulnerability. VP hyperactivation increases vascular tone, sodium retention, and sympathetic drive, while chronic CRH hypersecretion sustains hypothalamic–pituitary–adrenal (HPA) axis overactivity and systemic inflammation. These shifts converge in a “peptidergic collapse” of low OT/urocortins and high VP/CRH, linked to chronic inflammation, endothelial dysfunction, and outcomes such as stress cardiomyopathy, HFpEF, hypertension, and metabolic disorders. Loss of social buffering further amplifies these maladaptive pathways, undermining resilience.

## 9. Therapeutic Opportunities and Translational Frontiers

### 9.1. Peptidergic Agonists/Antagonists: VP Antagonists, OT Analogs, CRHR2 Activators

Targeting neuropeptidergic systems, including OT, VP, CRH, UCNs, represents a promising translational strategy across cardiovascular, neuropsychiatric, and metabolic domains. These systems link environmental stressors to internal regulatory responses, making them attractive targets for restoring autonomic balance, vascular function, and stress resilience.

#### 9.1.1. VP Antagonists: Mitigating Volume Overload and Sympathetic Drive

VP promotes vasoconstriction, water retention, and sympathetic activation, particularly in heart failure and hypertension. V1a and V2 receptor antagonists such as conivaptan and tolvaptan reduce preload and afterload by blocking vasoconstriction and renal water reabsorption. While currently approved for hyponatremia, they are under investigation for broader cardiovascular and neurocardiac applications [253]. Beyond diuresis, selective V1a antagonists may reduce sympathetic outflow and improve vagal balance, offering potential benefit in stress-related cardiovascular conditions. However, long-term outcomes and optimal dosing remain under investigation.

#### 9.1.2. OT Analogs: Enhancing Resilience and Autonomic Recovery

OT analogs and intranasal OT have emerged as modulators of stress and autonomic regulation. Experimental and early clinical studies show increased HRV, reduced blood pressure, and improved endothelial nitric oxide signaling following OT administration [92]. These effects are mediated in part through central actions on brainstem autonomic nuclei, enhancing parasympathetic output and dampening stress responses. The synthetic analog carbetocin, with a longer half-life and central-peripheral activity, is under investigation in neuropsychiatric and metabolic conditions and may have relevance for cardiometabolic regulation [278].

#### 9.1.3. CRHR2 Agonists and UCN Analogs: A Shift in Stress Pharmacology

While CRHR1 signaling is associated with stress pathology, CRHR2 activation, mediated by UCN2 and UCN3, exerts cardioprotective, vasodilatory, and anti-inflammatory effects. UCN2 improves cardiac output and reduces vascular resistance in preclinical heart failure models [204].

CRHR2 agonists enhance mitochondrial efficiency, increase HRV, and support endothelial function, positioning them as potential “hormetic mimetics” that activate adaptive stress pathways without the harmful effects of chronic CRH signaling. Their ability to recalibrate HPA axis activity and autonomic balance makes them promising candidates for complex, multisystem disorders.

### 9.2. Hormetic Conditioning Regimens (Intermittent Fasting, Thermal Stress)

Hormetic conditioning involves controlled exposure to low-dose stressors that activate adaptive physiological pathways. Interventions such as intermittent fasting, thermal stress, and exercise mimic evolutionary stress patterns and improve autonomic, metabolic, and vascular function.

#### 9.2.1. Intermittent Fasting and Nutrient Cycling

Intermittent fasting (IF) promotes metabolic switching from glucose to ketone utilization, improving mitochondrial efficiency, fatty acid oxidation, and antioxidant capacity. In both animal and human studies, IF improves endothelial function, reduces blood pressure, and increases HRV, indicating enhanced parasympathetic tone [279].

At the molecular level, IF activates AMPK, SIRT1, and PGC-1α while reducing oxidative stress and inflammation. These adaptations resemble those induced by exercise and are particularly beneficial in aging and cardiometabolic disease. IF may also influence OT and VP signaling, linking energy metabolism to neuroendocrine regulation.

#### 9.2.2. Thermal Stress: Sauna and Cold Exposure

Thermal stress, including heat exposure (sauna) and cold immersion, induces hormetic cardiovascular adaptations. Regular sauna use is associated with reduced cardiovascular mortality, improved endothelial function, and enhanced vagal tone. Heat exposure increases heat shock proteins, nitric oxide bioavailability, and vasodilation, improving microvascular function and arterial compliance [70].

Cold exposure induces transient sympathetic activation followed by parasympathetic rebound, enhancing autonomic flexibility. Repeated exposure promotes mitochondrial biogenesis, brown adipose tissue activation, and improved metabolic regulation. Both heat and cold interventions improve baroreflex sensitivity, vascular function, and inflammatory balance, supporting their use in conditions such as HFpEF and hypertension.

#### 9.2.3. Clinical Considerations and Future Directions

Individual variability in stress tolerance, health status, and neuroendocrine profile must guide implementation of hormetic interventions. Excessive or poorly timed exposure may shift adaptive responses toward maladaptation, particularly in vulnerable populations. Ongoing research is focused on optimizing dosing, frequency, and combined strategies, such as integrating fasting, thermal stress, and behavioral interventions, for safe and effective application.

### 9.3. Social and Affective Interventions (Touch, Group Therapy)

Social and affective interventions represent an underutilized therapeutic frontier in cardiovascular and neuroendocrine medicine. These approaches restore sociostasis by leveraging cues such as touch, empathy, proximity, and shared experience to modulate autonomic tone, reduce stress hormones, and engage neuropeptidergic systems including OT, VP, CRH.

#### 9.3.1. Touch and Physical Affiliation

Affective touch, including gentle stroking, hand holding, and massage, activates C-tactile afferents and promotes parasympathetic activity, reduced cortisol, and increased OT release [142]. Clinical studies show that structured caregiving and physical contact improve baroreflex sensitivity, HRV, and emotional regulation [52]. These findings indicate that social touch can restore autonomic balance and neurovisceral flexibility disrupted by chronic stress. Across the lifespan, from infancy to older adulthood, touch is associated with reduced HPA axis reactivity, enhanced oxytocinergic tone, and improved immune function.

#### 9.3.2. Group Therapy, Emotional Processing, and Shared Regulation

Group-based interventions, including support groups, psychodynamic therapy, and mindfulness-based stress reduction (MBSR), provide collective emotional regulation through shared experience and social validation. These approaches enhance vagal tone via synchronized affective engagement and co-regulation. Clinical studies demonstrate reductions in blood pressure, improved HRV, and decreased amygdala reactivity following group therapy [53]. These benefits are mediated not only by reduced isolation but also by modulation of OT–CRH signaling, particularly in individuals with trauma or chronic stress exposure.

#### 9.3.3. Mechanistic Insights: Peptidergic Mediation

Affective touch and shared emotional experiences increase endogenous OT, which reduces sympathetic activity, enhances vagal tone, and dampens HPA axis activation. These processes shift physiological regulation from threat-based to safety-oriented states. OT and VP also enhance empathy, trust, and social cognition, reinforcing feedback loops that sustain autonomic flexibility. CRH and UCN systems contribute by modulating arousal thresholds and facilitating recovery in socially safe environments.

#### 9.3.4. Translational Potential and Clinical Implications

Integrating social and affective interventions into cardiovascular care may significantly improve outcomes, particularly in individuals with high stress burden or social disconnection. These strategies are low-risk, cost-effective, and synergistic with pharmacologic and lifestyle therapies.

Future work should define optimal “dosing,” identify biomarkers of response such as salivary OT and HRV, and leverage digital tools to expand access to social interventions.

### 9.4. Biomarkers and Individualized Strategies

Incorporating neuroendocrine and autonomic biomarkers into clinical practice enables more precise assessment of stress-related cardiovascular risk. Traditional metrics such as blood pressure and ejection fraction do not capture physiological flexibility or stress reactivity, key components of resilience.

#### 9.4.1. Neuroendocrine Biomarkers

Salivary and plasma levels of OT, VP, CRH, and UCNs provide insight into stress activation and recovery. Higher OT levels are associated with improved vagal tone and reduced inflammation, while elevated VP and CRH correlate with endothelial dysfunction and autonomic imbalance [280].

#### 9.4.2. Physiological Biomarkers

HRV, baroreflex sensitivity, and flow-mediated dilation (FMD) offer dynamic, non-invasive measures of autonomic and vascular function. Reduced HRV and impaired FMD predict risk of HFpEF, Takotsubo cardiomyopathy, and arrhythmias independent of traditional risk factors [47]. Wearable technologies now enable continuous monitoring of these parameters.

#### 9.4.3. Mitochondrial and Inflammatory Markers

Markers of mitochondrial function, oxidative stress, and inflammation provide additional insight into stress adaptation. Indices such as mitochondrial health index (MHI) and inflammatory markers including IL-6, TNF-α, and CRP correlate with chronic stress exposure and cardiovascular risk [72].

#### 9.4.4. Toward Precision Medicine

Combining biomarker profiles with psychosocial factors enables individualized therapeutic strategies. For example, patients with low HRV and elevated VP/CRH may benefit from vagal stimulation or CRHR2-targeted therapies, whereas those with low OT and high inflammation may respond to social or OT-based interventions.

Future directions include composite biomarker panels, machine learning–based risk prediction, and sex- or trauma-informed approaches.

### 9.5. Intranasal OT and UCN Analogs in Therapy

Advances in peptide delivery have renewed interest in intranasal OT and UCN analogs as treatments for stress-related cardiovascular disorders. These agents target central and peripheral pathways involved in autonomic regulation, inflammation, and social buffering.

#### 9.5.1. Intranasal OT: Mechanisms and Applications

Intranasal OT reaches central autonomic regions via olfactory and trigeminal pathways, influencing the hypothalamus, amygdala, and brainstem. It enhances vagal tone and HRV, reduces HPA activity, and promotes anti-inflammatory and prosocial effects. In cardiovascular contexts, OT improves baroreflex sensitivity, reduces sympathetic activation, and enhances nitric oxide–mediated vasodilation. Intranasal carbetocin (LV-101) has demonstrated safety in clinical trials and is being explored for broader cardiometabolic applications [95,278].

#### 9.5.2. Urocortin Analogs and CRHR2 Activation

UCN2 and UCN3 activate CRHR2 receptors in the heart and vasculature, producing cardioprotective effects including improved contractility, vasodilation, reduced inflammation, and normalization of sympathetic tone. Preclinical studies show that UCN analogs reduce infarct size and improve cardiac function in HFpEF and stress cardiomyopathy models. However, clinical translation is limited by short half-life and delivery challenges. New formulations, including long-acting peptides, are under investigation [265,281].

#### 9.5.3. Precision Targeting and Safety

OT and UCN therapies exhibit dose- and context-dependent effects, requiring careful calibration to avoid adverse outcomes such as fluid retention or excessive vasodilation. Individual factors including sex, stress history, and receptor polymorphisms significantly influence therapeutic response, underscoring the need for personalized approaches.

##### Future Directions

Biomarker-guided trials combining OT/UCN analogs with autonomic and inflammatory profiling, Adjunct use in cardiac rehabilitation or post-myocardial infarction recovery, and Digital phenotyping and wearable tech to monitor acute responses in real-time. Together, intranasal OT and UCN analogs represent a promising class of neurovisceral therapeutics with potential to restore autonomic flexibility, social connectivity, and vascular resilience in high-risk patients.

### 9.6. Polyvagal Interventions and HRV Biofeedback

A hierarchical autonomic framework, articulated in Polyvagal Theory [27,111,153,186,189], has contributed to understanding how vagal pathways interface with social engagement and cardiovascular regulation in health and disease. This framework differentiates functionally distinct vagal circuits and highlights the role of myelinated vagal pathways in supporting states of calm engagement, metabolic efficiency, and adaptive recovery.

Importantly, this state-based perspective clarifies why identical stressors or therapeutic interventions may be adaptive in one physiological context yet maladaptive in another. Rather than viewing challenges as inherently beneficial or harmful, this model emphasizes the organism’s baseline autonomic organization, particularly the accessibility of ventral vagal cardioinhibitory regulation, as a determinant of physiological outcome [186]. Within this framework, experiences of safety and social connection reflect an autonomic configuration that supports efficient energy utilization, recovery, and plasticity. Hormetic responses are therefore most likely to emerge when challenges are encountered from within a state of regulatory flexibility, rather than during sustained sympathetic dominance or autonomic shutdown.

#### 9.6.1. Vagal Regulation and Cardioprotection

High cardiac ventral vagal tone, as indexed by RSA and HF-HRV, measures that reflect ventral vagal nucleus ambiguus–mediated cardioinhibitory regulation, is associated with lower resting heart rate, enhanced baroreflex sensitivity, reduced circulating inflammatory cytokines, and greater resilience to acute stress and emotional dysregulation [282,283]. In contrast, chronic stress, trauma, and social isolation are associated with diminished ventral vagal accessibility, autonomic rigidity, sustained sympathetic predominance, and increased cardiovascular risk [226]. Rather than serving as a singular therapeutic endpoint, RSA and HF-HRV may be understood as quantifiable indices of regulatory capacity within a hierarchically organized autonomic system, reflecting the organism’s ability to flexibly modulate cardiac output in response to environmental and social demands [258,282].

#### 9.6.2. HRV Biofeedback and Neuromodulation

HRV biofeedback trains individuals to regulate breathing patterns and interoceptive awareness in order to enhance cardiac vagal regulation and improve regulatory flexibility [282]. Clinical studies indicate that HRV biofeedback increases RSA and HF-HRV, reduces blood pressure and sympathetic arousal, improves mood, sleep quality, and emotional regulation, and may attenuate susceptibility to arrhythmia and ischemic events [284]. HRV biofeedback has shown promise in hypertension, heart failure, PTSD, and Takotsubo cardiomyopathy, particularly when integrated with pharmacologic and behavioral interventions [282,285]. Within the broader framework of hormesis, such approaches may expand the adaptive window through which physiological challenges are interpreted and metabolized, rather than simply “increasing vagal tone.”

#### 9.6.3. Brainstem-Targeted and Afferent-Based Interventions

Emerging interventions that engage vagal and related brainstem pathways include:

Transcutaneous auricular vagus nerve stimulation (taVNS), which delivers non-invasive stimulation to the auricular branch of the vagus nerve via external ear electrodes [285].

Acoustic-based interventions such as the Safe and Sound Protocol (SSP), which use filtered auditory input to influence brainstem-mediated regulation of auditory and autonomic circuits.

Rest and Restore Protocol (RRP) is a structured auditory implementation of Sonic Augmentation Technology™ that delivers dynamically modulated, off-grid acoustic input organized around endogenous autonomic time scales. Rather than pacing, synchronizing, or controlling physiological rhythms, RRP provides patterned sensory input designed to facilitate the expression of intrinsic brainstem- and autonomically mediated regulatory processes during periods of rest and recovery.

Touch-based therapies, including therapeutic massage or skin-to-skin contact, which activate C-tactile afferents associated with affiliative signaling and parasympathetic modulation [286].

These interventions are particularly relevant for cardiometabolic patients with histories of trauma, affective dysregulation, or social disconnection, populations that are often characterized by reduced cardiac vagal regulation and diminished peptide adaptability [106,287].

#### 9.6.4. Integration with Peptidergic and Hormetic Models

Afferent-based and brainstem-targeted interventions may complement hormetic conditioning strategies (e.g., structured exercise, thermal stress) and peptidergic therapies (e.g., intranasal OT or UCN analogs). Although mechanistically distinct, these approaches converge on a shared objective: enhancing adaptive regulatory capacity across autonomic, endocrine, and inflammatory systems [216,282,288]. Within the broader sociostasis–hormesis framework, such modalities may serve as non-pharmacologic entry points for influencing the brain–heart axis, particularly in individuals whose adaptive window has narrowed due to chronic stress or trauma [216,282,289].

## 10. Integrative Synthesis and Future Directions

### 10.1. Hormetic Sociostasis Model of Resilience

The hormetic sociostasis model proposes a unified conceptual framework in which physiological resilience and cardiovascular health emerge from the dynamic integration of hormetic stress responses and social homeostasis [51,290]. Rather than viewing stress as purely detrimental, this model emphasizes the necessity of intermittent, tolerable challenges, whether physical, emotional, or social, for maintaining adaptive capacity across multiple systems [210,291,292].

#### 10.1.1. Core Principles

Hormetic Stress as a Calibration Tool: Mild, intermittent stressors, such as exercise, fasting, thermal fluctuations, or cognitive challenge, induce a transient activation of molecular and cellular stress-response pathways (e.g., Nrf2, AMPK, SIRT1), leading to enhanced mitochondrial efficiency, vascular flexibility, and anti-inflammatory signaling [41].

Sociostasis as a Buffer and Amplifier: Human health depends not only on internal physiological regulation but also on social co-regulation. Close relationships, affective touch, and group belonging promote the release of OT and parasympathetic activation, mitigating the harmful effects of chronic stress while enhancing plasticity [293,294].

Peptidergic–Autonomic Integration: Neuropeptides like OT, VP, CRH, and UCNs function as key transducers that link psychosocial context with physiological reactivity. Their balance and timing determine whether stressors are processed as growth-inducing or health-eroding [210,295].

Dynamic Coupling Across Systems: Cardiovascular resilience arises from the synchronized regulation of the central autonomic network, baroreflex circuits, HRV-linked vagal pathways, and endothelial responses. This coupling is dynamically tuned by both environmental volatility and social safety signals and becomes impaired when either component, social buffering or hormetic adaptability, is disrupted [44].

#### 10.1.2. From Model to Practice

This integrative model offers a roadmap for intervention design across clinical, behavioral, and public health domains:

Pharmacologic targets: Peptide agonists/antagonists (e.g., CRHR2 agonists, intranasal OT).

Lifestyle interventions: Hormetic regimens (e.g., thermal therapy, fasting, low-dose ischemia).

Biobehavioral Regulatory Interventions: HRV biofeedback; acoustic and rhythm-based autonomic regulation protocols (e.g., Safe and Sound Protocol, Rest and Restore Protocol); somatic-oriented therapies including dance and structured movement; and social engagement or skills-based interventions.

Environmental design: Creating “safe spaces” that promote social signaling and stress recovery.

**Figure 14:**
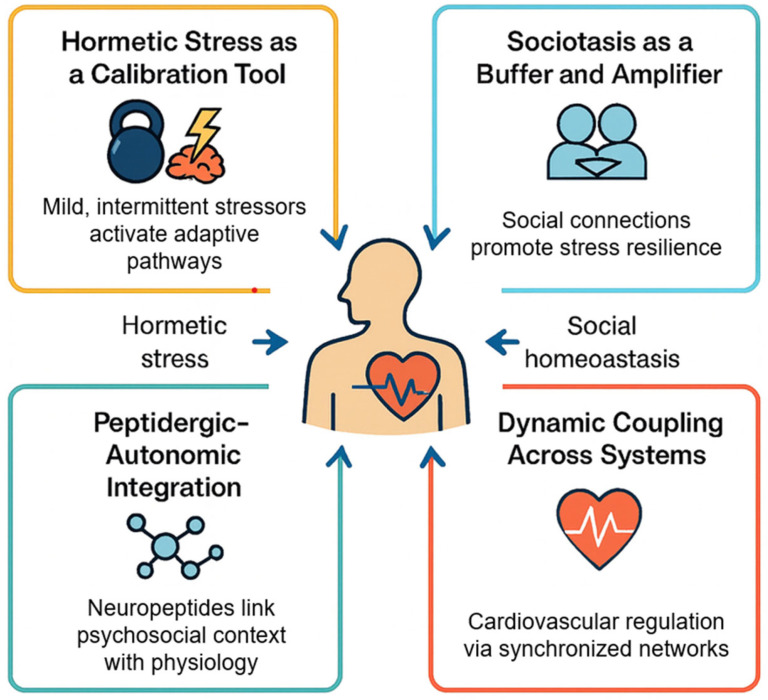
Hormetic Sociostasis Model of Resilience.

Rather than treating stress as an enemy, the hormetic sociostasis model recognizes it as a tool to be shaped, buffered, and balanced through the twin levers of adaptive challenge and relational safety (Figure 14).

This schematic shows how hormetic stress and sociostasis jointly drive resilience and cardiovascular health. Four domains are depicted: (1) hormetic stress from intermittent challenges activating adaptive pathways; (2) sociostasis through relationships and social belonging enhancing OT release and parasympathetic activity; (3) peptidergic–autonomic integration linking psychosocial context to physiology via oxytocin (OT), vasopressin (VP), and corticotropin-releasing hormone (CRH), urocortins (UCNs); and (4) dynamic coupling across systems, where autonomic, baroreflex, HRV, and endothelial responses synchronize to regulate cardiovascular function. Together, these mechanisms support resilience through adaptive challenges and social safety.

### 10.2. From Molecular to Interpersonal Adaptation

Understanding resilience in modern stress-related disease requires an integrative framework spanning molecular biology, neural systems, and social behavior. The convergence of hormesis, sociostasis, and neuropeptidergic regulation suggests that adaptation emerges not only within cells and circuits but also through interpersonal interactions.

#### 10.2.1. Molecular Roots of Resilience

At the molecular level, adaptation to intermittent stress engages conserved pathways including AMPK–SIRT1–PGC-1α signaling, Nrf2-mediated antioxidant responses, and heat shock protein networks. These mechanisms enhance mitochondrial function, redox balance, and autophagy, supporting cardiovascular and neural resilience [70]. Neuropeptides such as OT, VP, CRH, UCNs translate stress signals into coordinated physiological and behavioral responses, modulating inflammation, autonomic tone, and social cognition [210,295].

#### 10.2.2. Systems and Network Integration

These cellular processes scale to neural systems including the central autonomic network (CAN), limbic circuits, and social salience networks, which coordinate brain–heart interactions and behavior. OT and CRH–UCN systems act as regulatory hubs, suppressing threat responses in safe contexts while enabling adaptive mobilization under stress [216,227,247].

#### 10.2.3. Interpersonal Co-Regulation

At the interpersonal level, social cues such as touch, synchrony, and supportive relationships regulate physiology. Caregiving, affective touch, and group affiliation enhance vagal tone, reduce sympathetic load, and promote adaptive neuroplasticity [286,296].

These interactions are biologically embedded mechanisms of resilience, largely mediated by OT and vagal pathways. Thus, resilience is both cellular and social, emerging from interactions between molecular systems and relational environments.

#### 10.2.4. Clinical and Translational Implications

Psychosocial interventions such as group therapy, affective touch, and caregiver training should be viewed as biological modulators rather than adjunctive support. Integrated approaches may combine pharmacologic strategies, such as CRHR2 agonists or intranasal OT, with biobehavioral interventions including HRV biofeedback and vagal regulation techniques. Multilevel biomarkers capturing mitochondrial function, peptide activity, autonomic dynamics, and social context may guide personalized treatment strategies. This framework reframes resilience as a trans-scalar property shaped by both biology and environment.

### 10.3. Personalized Medicine and Neurocardiac Biomarkers

Recognition of individual variability in stress responses and cardiovascular vulnerability has accelerated the shift toward personalized medicine. Effective approaches require integration of molecular, neural, autonomic, and social biomarkers to enable early detection and targeted intervention [297,298].

#### 10.3.1. Neurocardiac Biomarkers of Resilience and Risk

Key biomarkers at the brain–heart interface include:

- HRV as a measure of vagal tone and regulatory flexibility
- OT levels reflecting social buffering capacity
- CRH and UCN activity indicating stress response patterns
- Mitochondrial health indices assessing bioenergetic function
- Inflammatory markers such as IL-6, TNF-α, and CRP

Combined, these metrics differentiate adaptive hormetic responses from maladaptive allostatic load and enable dynamic risk stratification.

#### 10.3.2. Multi-Omics and Digital Phenotyping

Integration of genomic, epigenomic, transcriptomic, and peptidomic data enhances precision. Genetic variants in OXTR, AVPR1A, or CRHR2 may influence stress sensitivity and treatment response, while epigenetic changes in stress-related genes link early adversity to disease risk. Emerging tools such as saliva-based miRNAs and wearable technologies capturing HRV, sleep, and activity allow continuous, real-world physiological monitoring, further refining individualized assessment.

#### 10.3.3. Tailoring Interventions

Personalized strategies may include:

- Peptide-based therapies such as intranasal OT or CRHR2 agonists for specific neuroendocrine profiles
- HRV biofeedback and vagal-targeted interventions for impaired autonomic regulation
- Hormetic regimens including fasting, thermal stress, or exercise tailored to metabolic and autonomic status
- Social and affective interventions for individuals with reduced social buffering capacity
- Machine learning applied to multimodal datasets may further improve prediction, treatment selection, and monitoring of therapeutic response.

### 10.4. Open Research Questions and Emerging Technologies

Despite rapid advances in understanding the neuroendocrine, autonomic, and behavioral mechanisms linking stress to cardiovascular health, several critical questions remain unresolved. Addressing these gaps requires novel tools, longitudinal frameworks, and cross-disciplinary collaboration that spans molecular neuroscience, cardiology, behavioral science, and computational modeling.

#### 10.4.1. Unanswered Questions in Peptidergic Cardiometabolic Regulation

Temporal dynamics of peptide interaction: How do OT, VP, CRH, and UCNs interact across different phases of acute and chronic stress in humans? While animal models suggest temporal gating, translational evidence in clinical populations is lacking.

Sex differences and hormonal modulation: To what extent do gonadal hormones shape peptidergic tone and vulnerability to stress-related cardiovascular diseases? The interplay between estrogen/testosterone and OT/CRH–UCN axes remains underexplored.

Dose–response thresholds for hormetic interventions: What is the optimal intensity, frequency, and duration of intermittent fasting, heat/cold exposure, or exercise to elicit protective rather than maladaptive responses in different populations (e.g., elderly, hypertensive, post-MI)?

Mechanisms of social buffering: What specific neural and peripheral pathways mediate the cardioprotective effects of touch, affiliation, and group cohesion? Can these pathways be pharmacologically or digitally enhanced?

Biotype classification and responder phenotyping: Can we define biologically distinct “stress-coping” phenotypes based on neurocardiac biomarkers and sociobehavioral traits to better target treatments?

#### 10.4.2. Emerging Technologies to Address These Gaps

Single-cell multi-omics and spatial transcriptomics: Enable precise mapping of neuropeptide receptor expression (OTR, AVPR1A, CRHR2) in stress-related brain and cardiac tissues, uncovering cell-type–specific regulatory networks.

Next-generation biosensors and neurocardiac wearables: Advances in wearable technology, measuring HRV, skin conductance, sleep architecture, and social interactions, allow for high-resolution temporal tracking of real-world stress responses.

Intranasal delivery systems and engineered peptides: Novel formulations and delivery vectors (e.g., nanoparticle-based, self-assembling peptides) are improving the bioavailability and targeting of OT and UCN analogs in human trials.

Artificial intelligence and digital twin modeling: Machine learning algorithms can synthesize multimodal datasets (e.g., genomics, HRV, imaging, behavior) to predict individual disease trajectories and therapeutic responsiveness. “Digital twins” of patient neurocardiac systems could simulate responses to interventions.

Closed-loop neuromodulation systems: Devices such as transcutaneous vagus nerve stimulators and HRV-biofeedback platforms may be optimized using adaptive algorithms that respond to real-time autonomic or behavioral feedback.

##### The Road Ahead

The integration of systems biology, digital medicine, and neuropeptide pharmacology holds transformative potential. To fully realize a precision stress medicine framework, future research must link real-time physiological monitoring with targeted modulation of stress–sociostasis circuits. The long-term goal is not merely the prevention of cardiovascular disease, but the cultivation of adaptive resilience, rooted in both biology and social connection.

## 11. Conclusions

The integration of sociostasis and hormesis offers a novel and unifying perspective on cardiovascular health. While traditional models of heart disease have emphasized mechanical and metabolic factors, this review underscores the importance of adaptive neuroendocrine, autonomic, and behavioral regulation in maintaining cardiovascular resilience. Sociostasis, the dynamic balance of social connection and neurobiological regulation, serves as a protective scaffold against stress-induced physiological dysregulation. Hormesis, in turn, highlights the paradoxical benefits of intermittent, low-level challenges that recalibrate systemic defenses and optimize stress responsiveness.

Central to this integrative model is the OT–VP–CRH–UCN neuropeptidergic network, which modulates the interplay between social experience, stress physiology, and cardiovascular function. These peptides not only coordinate the internal milieu in response to threat but also mediate the reparative and affiliative processes necessary for recovery and resilience. Their bidirectional communication between the brain and heart, especially via the ANS and inflammatory pathways, represents a crucial frontier in cardiovascular science.

Furthermore, the failures of these systems, whether due to chronic stress, social disconnection, or maladaptive plasticity, underpin a wide spectrum of disorders, including hypertension, heart failure, Takotsubo syndrome, and metabolic syndrome. Conversely, therapeutic strategies that target these systems, ranging from peptidergic agonists, intermittent conditioning regimens, and intranasal neuropeptides to social-touch therapies and HRV-guided neuromodulation, demonstrate potential to restore balance and resilience. As the field moves toward precision cardiovascular medicine, it must embrace multilevel models that incorporate the molecular, autonomic, emotional, and social dimensions of heart health.

Future research should not only elucidate the mechanisms by which stress and social context shape cardiovascular outcomes but also develop interventions that enhance the flexibility, adaptability, and coherence of the brain–heart axis. The heart, in this view, is not just a pump, but a socially responsive, neuroendocrine organ, capable of learning, adapting, and healing through its connections with the brain and others. Integrating sociostasis and hormesis may thus provide more complete blueprint for fostering resilience in both individuals and populations.

## Figures and Tables

**Table 1:** Guiding questions framing the conceptual scope of the present review.

Guiding Questions	Conceptual Scope
How does the social environment influence cardiovascular physiology at the molecular and systemic level?	Examines social determinants of cardiovascular health, including relational support, isolation, and socio-environmental stressors, at both molecular (gene expression, signaling pathways) and systemic (autonomic, hemodynamic) levels.
What are the roles of OT, VP, CRH, and UCNs in shaping cardiovascular responses to acute and chronic stress?	Explores the mechanistic roles of OT, VP, CRH, and UCNs in modulating cardiovascular reactivity and recovery across different stress contexts.
How does exposure to low dose, manageable stress (hormesis) initiate protective adaptations in the cardiovascular system?	Investigates how mild, intermittent stressors trigger adaptive responses such as enhanced baroreflex sensitivity, improved vascular compliance, and anti-inflammatory signaling.
What are the consequences of chronic, unbuffered stress on the balance of neuropeptidergic and autonomic regulation?	Analyzes how prolonged stress without adequate buffering disrupts the equilibrium between neuropeptidergic systems and autonomic control, leading to heightened allostatic load and cardiovascular risk.
Can these insights be translated into preventive, diagnostic, or therapeutic innovations for cardiovascular disease?	Assesses translational opportunities for biomarker discovery, targeted neuropeptide-based therapeutics, and integrated prevention strategies for stress-related cardiovascular disorders.

## Data Availability

No new data were created or analyzed in this study. Data sharing is not applicable to this article.

## Abbreviations

ACTH	adrenocorticotropic hormone
AMPK	AMP-activated protein kinase
ANS	Autonomic nervous system
AREs	Antioxidant response elements
ATP	Adenosine triphosphate
BNST	bed nucleus of the stria terminalis
CRH	Corticotropin-releasing hormone
DMNX	Dorsal motor nucleus of the vagus
eNOS	Endothelial nitric oxide synthase
FOXO	Forkhead box O
FMD	Flow-mediated dilation
GPx	glutathione peroxidase
HF-HRV	High-frequency heart rate variability
HFpEF	Heart failure with preserved ejection fraction
HO-1	Heme oxygenase-1
HPA axis	Hypothalamic–pituitary–adrenal axis
HRV	Heart rate variability
HSPs	Heat shock proteins
IF	Intermittent fasting
IPC	Ischemic preconditioning
K_ATP	ATP-sensitive potassium channel
Nrf2	Nuclear factor erythroid 2-related factor 2
NO	Nitric oxide
NQO1	NAD(P)H quinone oxidoreductase 1
NTS	Nucleus tractus solitarius
OT	Oxytocin
OTR	Oxytocin receptor
OXTR	Oxytocin receptor genes
PGC-1α	Peroxisome proliferator-activated receptor gamma coactivator 1-alpha
PFC	Prefrontal cortex
PI3K/Akt	Phosphoinositide 3-kinase/Protein kinase B
PKC	Protein kinase C
PTSD	Post-traumatic stress disorder
PVN	Paraventricular nucleus
RIC	Remote ischemic conditioning
ROS	Reactive oxygen species
RSA	Respiratory sinus arrhythmia
SIRT1/3	Sirtuin 1 and Sirtuin 3
SOD	Superoxide dismutase
UCNs	Urocortins
VE	Vagal efficiency
VP	Vasopressin
V1a	Vasopressin receptor type 1a
